# Discovery proteomics defines androgen-regulated glycoprotein networks in prostate cancer cells, as well as putative biomarkers of prostatic diseases

**DOI:** 10.1038/s41598-021-01554-2

**Published:** 2021-11-15

**Authors:** Jordy J. Hsiao, Melinda M. Smits, Brandon H. Ng, Jinhee Lee, Michael E. Wright

**Affiliations:** Department of Molecular Physiology and Biophysics, Carver College of Medicine, The University of Iowa, Iowa City, IA 52242 USA

**Keywords:** Prostate cancer, Proteomics

## Abstract

Supraphysiologic androgen (SPA) inhibits cell proliferation in prostate cancer (PCa) cells by transcriptional repression of DNA replication and cell-cycle genes. In this study, quantitative glycoprotein profiling identified androgen-regulated glycoprotein networks associated with SPA-mediated inhibition of PCa cell proliferation, and androgen-regulated glycoproteins in clinical prostate tissues. SPA-regulated glycoprotein networks were enriched for translation factors and ribosomal proteins, proteins that are known to be *O*-GlcNAcylated in response to various cellular stresses. Thus, androgen-regulated glycoproteins are likely to be targeted for *O*-GlcNAcylation. Comparative analysis of glycosylated proteins in PCa cells and clinical prostate tissue identified androgen-regulated glycoproteins that are differentially expressed prostate tissues at various stages of cancer. Notably, the enzyme ectonucleoside triphosphate diphosphohydrolase 5 was found to be an androgen-regulated glycoprotein in PCa cells, with higher expression in cancerous versus non-cancerous prostate tissue. Our glycoproteomics study provides an experimental framework for characterizing androgen-regulated proteins and glycoprotein networks, toward better understanding how this subproteome leads to physiologic and supraphysiologic proliferation responses in PCa cells, and their potential use as druggable biomarkers of dysregulated AR-dependent signaling in PCa cells.

## Introduction

In the United States, 174,650 new cases of prostate cancer (PCa) were diagnosed and 31,620 PCa-related deaths were reported in 2019, making PCa the second leading cause of cancer-related deaths of men^1^. Localized PCa is a curable disease, with a 5-year relative survival rate of nearly 100%. This is in stark contrast to the survival rate for metastatic PCa, which is 33%^1^. One reason for this difference is that long-term androgen-deprivation therapy (ADT), which is commonly used for the treatment of hormone-naïve metastatic PCa, selects for the outgrowth of ADT-resistant tumors called castrate-resistant prostate cancer (CRPC). Most forms of PCa are consequences of dysregulated signaling by the androgen receptor (AR), a powerful sex steroid hormone receptor (SHR) that regulates the transcription of genes governing the proliferation, survival, and differentiation of both normal and neoplastic prostate epithelial cells^2^. Aberrant AR-dependent transcription is a conserved feature of localized PCa, metastatic PCa (mPCa), and CRPC. For example, in the case of localized PCa, approximately 50% of patients of Caucasian/Eastern European-descended populations harbor gene rearrangements that place transcription factors of the oncogenic ETS family under the control of the androgen-regulated promoter element *TMPRSS2*^3^. These *TMPRSS2-ETS* gene fusions promote ETS overexpression, increase tumor cell motility and invasiveness, and induce prostatic intraepithelial neoplasia in genetically engineered mice. Moreover, when these fusions are combined with *Pten* loss or increases in AR signaling, they increase the invasiveness of prostate carcinomas^4–7^. Similarly, CRPC involves the dysregulation of AR-dependent signaling by a variety of causes, including: AR mutations, AR amplification, AR variants, aberrant expression of AR coregulators, androgen synthesis by tumors^8,9^, and amplification of upstream enhancers of *AR*^10–12^. These defects all enable the tumor cells to bypass the actions of ADT and second-generation AR pathway inhibitors (ARPIs) (*i.e.*, enzalutamide and abiraterone)^13–15^. Moreover, sustained targeting of AR with ARPIs promotes divergent clonal evolution to neuroendocrine (NE)-CRPCs^16^, in which AR expression is downregulated and growth and survival are driven by AR-independent mechanisms^17–19^.

Strategies for the treatment of CRPC have focused on attenuating aberrant AR activity in tumor cells^8^ and exploiting epigenetic pathways to re-sensitize NE-CRPC to ARPIs through the re-expression of AR^18^. Recent efforts have explored the use of supraphysiological androgen (SPA) in the treatment of PCa^20^ because high doses of androgens inhibit the proliferation of AR-positive PCa cells^21–26^. Genomic technologies have pioneered the discovery of androgen-regulated genes (ARGs) and sought to determine how the aberrant expression of such genes contributes to the development and progression of localized PCa, mPCa, and CRPC^4,27–29^. In AR-positive PCa cells, proliferation is stimulated by physiologic concentrations androgens (PA) (*e.g.,* 0.1–1 nM dihydrotestosterone-DHT) and repressed by SPA (*e.g.,* ≥ 10 nM DHT or ≥ 1n nM synthetic androgen R1881)^24,26,30–39^. SPA antagonizes cell proliferation by causing AR-mediated repression of both cell-cycle genes (*e.g.*, cyclin D1, CDK4/6, CDKN1A)^40^, DNA replication genes (*e.g.*, MCM4)^41^, and genes linked to cellular senescence^38^. Although the transcriptional mechanisms underlying proliferation inhibition in PCa cells by SPA are well-defined, how SPA influences protein networks (*i.e.*, what the effects on the proteome are) has yet-to-be established.

In this study, we used proteomic profiling to identify androgen-regulated glycoproteins and glycoprotein networks that are linked to SPA-mediated inhibition of proliferation of the LNCaP PCa cell line. We discovered that androgens elicited dose-dependent changes in the expression of glycoprotein networks related to cell growth and differentiation. Lastly, the glycoproteomic dataset represents a rich source of information about how androgen-regulated protein glycosylation relates to androgen-mediated proliferation responses in PCa cells and, more generally, will be useful for interrogating the function of androgen-regulated glycoproteins in prostatic diseases.

## Results

### Glycoprotein networks associated with androgen-mediated proliferation responses in cells of the human LNCaP line

We set out to understand how androgen-mediated proliferation responses are coupled to changes in protein glycosylation in PCa cells, in particular, how androgen levels influence changes in the glycosylation of growth factor receptors that harbor *N*- and *O*-linked oligosaccharides or intracellular proteins that contain the *O*-linked N-acetylglucosamine (*O*-GlcNAc) moiety^42,43^. The LNCaP cell line, which models PCa, was selected for the glycoprotein profiling experiment because the inhibition of SPA-mediated proliferation in this context is well-documented^30^. As shown previously^21–23^, physiologic levels of androgen (*i.e.*, 1 nM R1881) stimulated maximal proliferation of LNCaP cells, whereas SPA (*i.e.*, 10 nM R1881) attenuated their proliferation (Supplemental Fig. 1A,B). These findings showed that androgenic responses in LNCaP cells are biphasic and dose-dependent, justifying their use as an experimental model for studying how the inhibition of SPA-mediated proliferation influences protein glycosylation in PCa cells.

**Figure 1:**
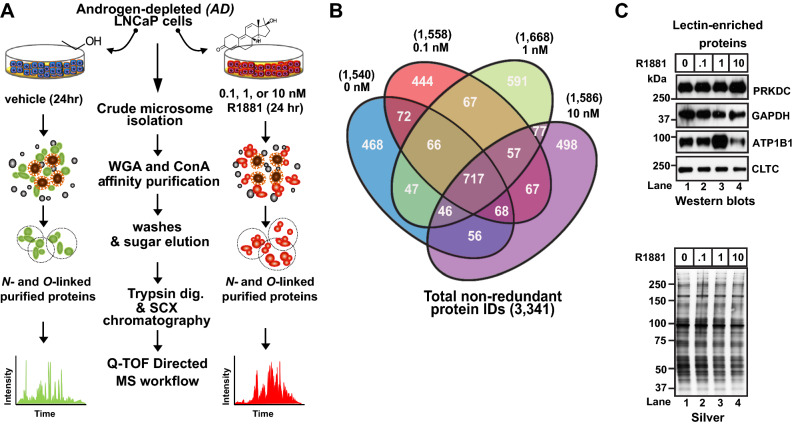
Quantitative mass spectrometry profiling of membrane-associated glycoproteins in LNCaP cells. (**A**) Experimental workflow for the proteomic profiling of *N*-linked and *O*-linked glycosylated membrane proteins expressed in LNCaP following exposure to androgen-depleted (*AD*) and androgen-stimulated (*AS*) (*i.e.*, 0.1 nM, 1 nM, or 10 nM R1881) growth medium. (**B**) Summary of protein quantification analysis. Venn diagram illustrating quantities of proteins shared among and unique to vehicle (*i.e.*, EtOH) and androgen-treated (*i.e.*, 0.1, 1, and 10 nM R1881) samples. A total of 3,341 non-redundant proteins were identified as being differentially expressed, and they were quantified across all samples. (**C**) Semi-quantitative western-blot showing expression of DNA-dependent protein kinase catalytic subunit (PRKDC), glyceraldehyde 3-phosphate dehydrogenase (GAPDH), sodium and potassium ATPase (Na + /K + ATPase), and clathrin heavy chain 1 (CLTC) in glycoprotein-enriched extracts. (Lower panel) Silver-stain gel showing loading of glycoprotein-enriched samples.

To identify SPA-mediated changes in protein glycosylation, we prepared crude microsomes from 72 h androgen-starved LNCaP cells and challenged them with subphysiologic androgen (*i.e.,* 0 nM R1881), physiologic androgen (PA) (*i.e.,* 0.1 and 1.0 nM R1881), or SPA (Fig. 1A) for 24 h. These detergent-solubilized microsomes were then subjected to lectin weak affinity chromatography (LWAC)^44^ to enrich for glycosylated membrane and membrane-associated proteins. LWAC was performed using a mixture of wheat-germ agglutinin-(WGA) and concanavalin A-(ConA) conjugated sepharose beads, to capture both *N*-linked (*i.e.,* asparagine-linked *N*-acetyl glucosamine-GlcNAc, serine/threonine-linked GlcNAc, and sialic acid) and *O*-linked (*i.e.,* serine and threonine) glycoproteins (α-linked mannose, terminal glucose moieties) (Fig. 1A). Lectin-enriched protein samples from the LNCaP cells were subjected to label-free protein identification and quantification via directed MS (dMS)^45^ (Fig. 1A). This glycoproteomic profiling experiment resulted in the quantification of 3,341 non-redundant proteins (IDs) across all experimental conditions. The number of IDs obtained using each concentration of R1881 was: 1540 (0 nM), 1,558 (0.1 nM), 1,668 (1.0 nM), and 1,586 (10 nM) (Fig. 1B, Supplemental Table I).

As an orthogonal approach to verify the observed changes in protein expression, we used semi-quantitative western blots. Proteins were selected for verification based upon the commercial availability of western blot-grade antibodies. They were DNA-dependent protein kinase catalytic subunit (PRKDC), glyceraldehyde-3-phosphate dehydrogenase (GAPDH), sodium/potassium-transporting ATPase subunit beta-1 (ATP1B1), and clathrin heavy chain 1 (CLTC) (Fig. 1C, Supplemental Fig. 2). With the exception of PRKDC at 10 nM R1881, western blots across the doses of androgen tested were roughly concordant with protein abundance changes as determined by dMS (Table 1). These findings showed that the glycoproteomic profiling experiment has the power to detect changes in the expression of microsomal glycoproteins in androgen-treated LNCaP cells.

**Table 1:** Spectrum mill protein quantification.

0 nM R1881	0.1 nM R1881	1.0 nM R1881	10.0 nM R1881	UniProt accession number	Description	Gene name
3.72E+07	2.71E+07	3.29E+07	2.44E+07	P78527	DNA-dependent protein kinase catalytic subunit	PRKDC
9.66E+07	9.03E+07	2.48E+07	3.06E+07	P04406	Glyceraldehyde-3-phosphate dehydrogenase	GAPDH
8.18E+06	4.97E+06	1.40E+07	2.86E+05	P05026	Sodium/potassium-transporting ATPase subunit beta-1	ATP1B1
2.99E+08	1.93E+08	1.95E+08	1.43E+08	Q00610	Clathrin heavy chain 1	CLTC

Total protein abundance after samples were normalized to externally spiked bovine serum albumin.

**Figure 2:**
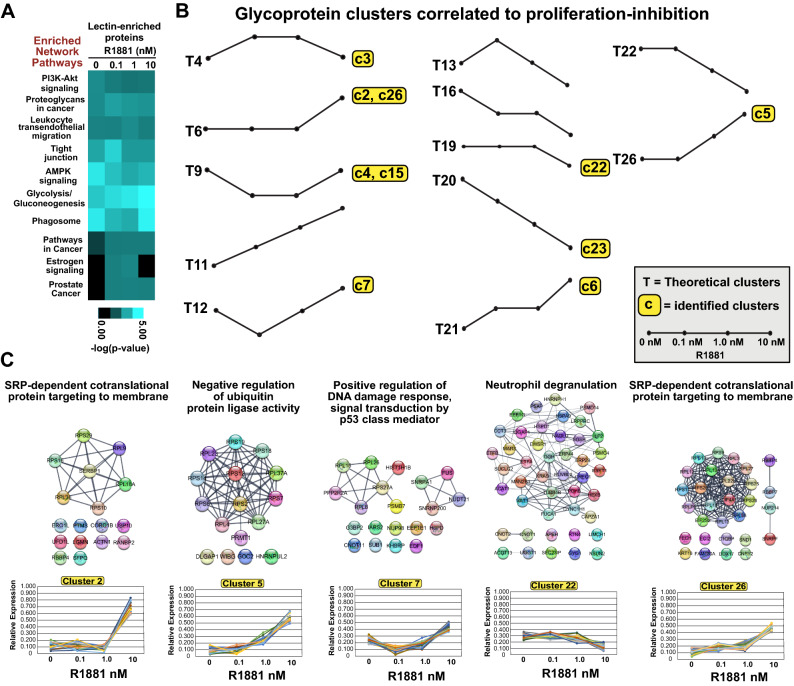
Bioinformatic analyses of glycoproteome. (**A**) Heat-map visualization of the ten top-ranked glycoprotein pathways in androgen- vs. vehicle-treated samples. The WebGestalt bioinformatics program and KEGG pathway analysis were used. Enriched network pathways and computed *p*-values < 0.05 are reported as –log (*p*-value). Data were transformed using the TreeView software. (**B**) Theoretical clusters and clusters identified in the context of growth inhibition at 10 nM androgen. Of 27 theoretical protein clusters, 10 were found to be increased or decreased in samples treated with 10 nM vs. 1 nM R1881-treated samples. (**C**) STRING protein–protein interactions in six clusters of proteins (*i.e.*, cluster 2, 5, 7, 14, 22, and 26) enriched for biological processes as determined by the WebGestalt overrepresentation enrichment analysis (OEA) program. Protein interaction networks were constructed and visualized in Cytoscape using the STRING plug-in application. Each cluster is annotated with the top-ranked enriched biological process above its PPI plot.

To identify molecular associations between the androgen-mediated proliferation responses in LNCaP cells and specific biological pathways, we evaluated the enrichment and de-enrichment of glycoproteins across treated samples. Biological pathways that were over- or under-represented across the tested androgen concentrations were identified using the WEB-based Gene SeT AnaLysis Toolkit (WebGestalt) program (Fig. 2A). Notably, ten of the top-ranked biological pathways were conserved across each experimental sample (*i.e.,* 0, 0.1, 1.0, 10 nM R1881), highlighting similarities in the molecular composition of lectin-enriched proteomes profiled in the glycoproteomic experiment (Fig. 2A). TreeView visualization of lectin-enriched proteomes shows differences in the enrichment of biological pathways in experimentally-treated samples (Fig. 2A)^46^. The biological pathways included PI3K-Akt signaling, Proteoglycans in cancer, Leukocyte transendothelial migration, Tight junctions, AMPK signaling, Glycolysis/Gluconeogenesis, Phagosome, Pathways in cancer, Estrogen signaling, and Prostate cancer (Fig. 2A). Despite conservation of the biological pathways, different clusters of protein–protein interactions (PPIs) were detected at each dose of androgen, highlighting potential differences in the glycosylation status of PPIs (Supplemental Fig. 3). Importantly, androgens caused dose-dependent changes in the expression of components of specific biological pathways. For example, components of the Glycolysis/Gluconeogenesis pathway were more commonly expressed at higher doses of androgen (Fig. 2A). The opposite effect was observed for the PI3K-Akt pathway, with components less commonly expressed at higher doses of androgen. In contrast, in the cases of the AMPK, Phagosome, and Estrogen signaling pathways, responses were biphasic. Levels of expression of components of both the AMPK and Phagosome pathways were reduced at physiologic levels of androgen but were reduced in the context of SPA (10 nM R1881). The Estrogen signaling pathway showed an inverse biphasic response to androgens, with signaling elements increased at physiologic androgen levels but reduced at SPA. Lastly, signaling components of the Tight junction and Leukocyte transendothelial migration pathways were selectively increased at physiologic levels of androgen, suggesting that these pathways are sensitive to a narrower range of androgens. Overall, these results show that in LNCaP cells the glycosylated protein content of membranes and the representation of specific biological pathways are regulated by androgens in LNCaP cells.

These findings prompted us to identify glycoproteins that are androgen-coregulated in response to SPA, because they could represent specific biomarkers of androgen-mediated inhibition of cell proliferation. To this end, we subjected our samples to empirical *K*-means clustering and assessed glycoprotein clusters at each dose of androgen. We generated 27 glycoprotein clusters based upon the assumption that such clusters could either increase, decrease, or remain unchanged at each concentration of androgen (*i.e.,* factorial calculation 3^3^ = 27 clusters) (Supplemental Figs. 4 and 5, Supplemental methods). Our goal was to identify glycoprotein clusters that were (1) concordant at the SPA and 0 nM dose of androgen, i.e., that are associated with reduced proliferation of LNCaP cells and (2) discordant at the 0.1 nM and 1.0 nM doses of androgen, both of which stimulated the proliferation of LNCaP cells. Only twelve of the 27 theoretical clusters fit this selection criterion (*i.e.,* clusters 4, 6, 9, 11, 12, 13, 16, 19, 20, 21, 23, and 26) (Fig. 2B) (Supplemental Fig. 6). Moreover, only ten clusters (*i.e.*, c2-c7, c15, c22, c23, and c26) from our dataset matched the twelve theoretical clusters (*i.e.*, T4, T6, T9, T12, T19, T20, T21, and T26) (Fig. 2B) (Supplemental Fig. 6). Given that the eight glycoprotein clusters were associated with androgen-mediated inhibition of proliferation, we tested glycoprotein clusters for functional relationships with a specific biological process in response to SPA. WebGestalt analyses identified overrepresented biological pathways in five of the eight glycoprotein clusters (*i.e.*, c2, c5, c7, c22, and c26) (Fig. 2C) (Supplemental Excel file 2). The biological pathways included SRP-dependent cotranslational protein targeting to membrane (*i.e.,* clusters 2 and 26), Negative regulation of ubiquitin protein ligase activity (*i.e.,* cluster 5), Positive regulation of DNA damage response-p53 class mediator, and Neutrophil degranulation (Fig. 2C). These results suggest that SPA-mediated inhibition of proliferation is associated with the coordinated glycosylation of functional protein complexes and their underlying specific biological pathways in LNCaP cells.

### ENTPD5 is an androgen-regulated glycoprotein in LNCaP cells and is differentially expressed in clinical prostate tissue

**Figure 3:**
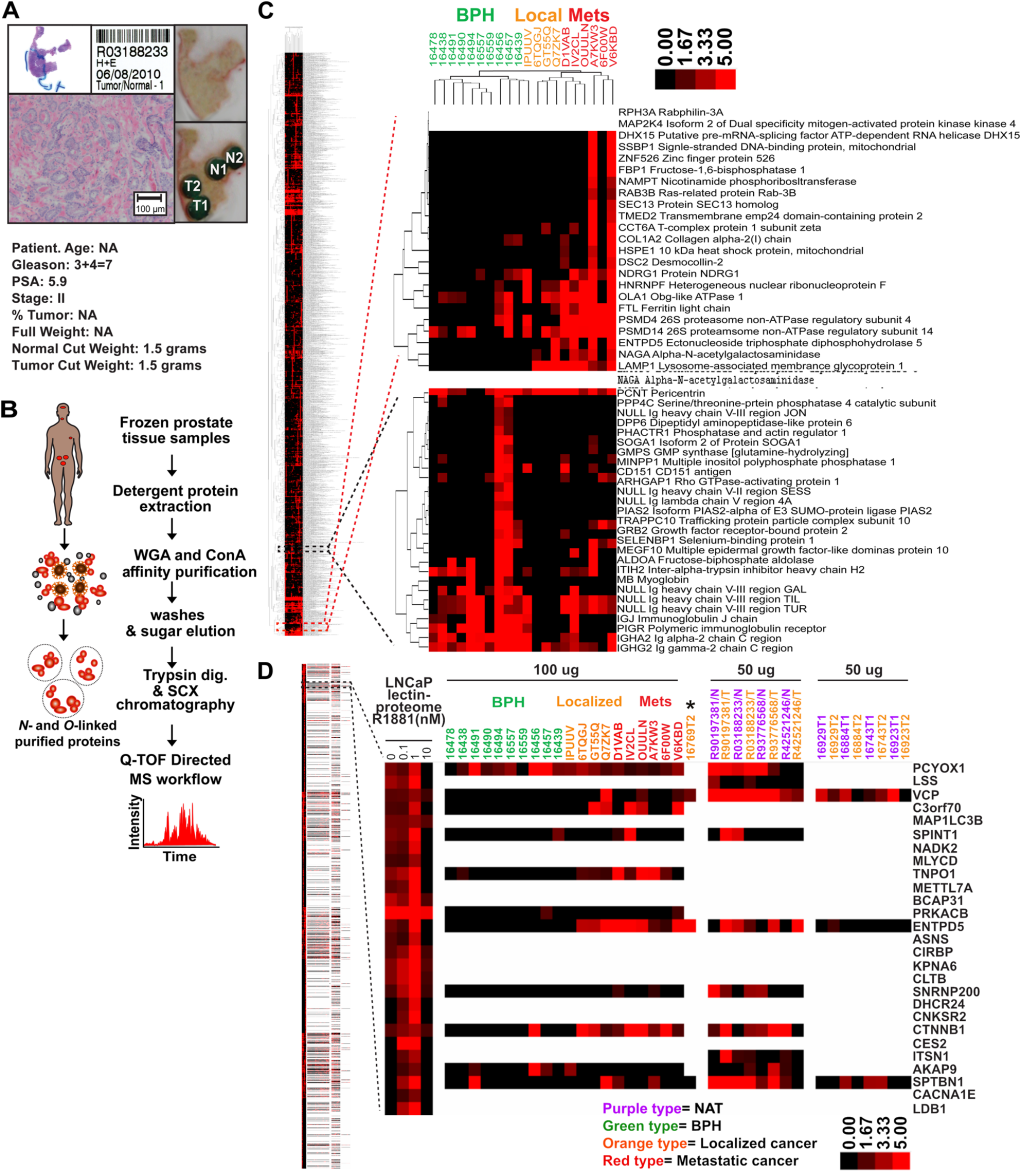
Glycoproteomic workflow for clinical prostate tissues. (**A**) Resection of tumor tissue (TT) and normal adjacent tissue (NAT) from clinical prostate tissue samples to be used in glycoproteomic mass spectrometry analyses. H&E slide of tissue sample, light microphotographs of resected regions of TT and NAT, clinical characteristics of patient data, and physical properties of resected tissue samples. (**B**) Summary of glycoproteomic workflow used to extract glycoproteins from frozen prostate tissue samples. **(C)** Hierarchical clustering of androgen-sensitive glycoproteins and clinical prostate tissues. Protein expression in BPH and in localized PCa and metastatic PCa samples (*i.e.*, 100 μg of protein was processed for dMS analyses) after normalization to externally spiked BSA. 1,759 expressed proteins across 20 tissue samples (*i.e.*, 10 BPH, 4 localized PCa, and 6 metastatic PCa) were clustered using Pearson correlation distances between averages using GenePattern. Magnified clusters exemplify those with increased protein expression in cancer vs. BPH samples (*i.e.*, top right panel) and those with increased protein expression in BPH vs. cancer samples (*i.e.*, bottom right panel). Expression scale for fold expression was 0 (*i.e.*, black color, undetectable expression) up to 5 (*i.e.*, bright red, saturated expression). (**D**) Cluster analysis of glycoproteins in LNCaP cells, BPH, localized PCa and metastatic PCa tissue normalized to externally spiked BSA. The glycoproteins in LNCaP cells (*i.e.*, 0, 0.1, 1, and 10 nM R1881-treated cells) and glycoproteins from BPH, localized PCa, and metastatic PCa samples (*i.e.*, 37 tissue samples, including: 10 BPH; 5 localized PCa; 6 metastatic PCa -100 μg and 8 paired TT/NAT samples- 50 μg) were clustered using Pearson correlation distances between averages using GenePattern. Magnified cluster represents ENTPD5 expression in LNCaP cells and metastatic tissue samples. The expression scale for the clustergram denotes the relative protein abundance, ranging from 0 for no protein expression (black) to saturation (red) at fivefold expression. Clustered androgen-regulated glycoproteins in LNCaP cells whose expression increased in metastatic tissue samples are highlighted in the magnified clustergram.

We sought to leverage the glycoproteomic profiling strategy to analyze clinical prostate tissues for differences in glycoprotein expression between BPH, localized PCa, and metastatic PCa samples and, where available, paired NAT. A total of 37 fresh-frozen prostate tissue samples, which represented 29 cases (8 NAT, 10 BPH, 13 localized PCa, and 6 mPCa samples), were collected and processed for the glycoproteomic profiling experiments (Supplemental Table II–IV) (Fig. 3) (Supplemental Figs. 7–9). Our initial glycoproteomic profiling experiments of PCa tissues and paired NAT (*i.e.,* R90197381-N/T, R42521246-N, R93776568-N/T, R03188233-N/T) involved extensive peptide fractionation using strong cation exchange (SCX), high-performance liquid chromatography (HPLC) (*e.g.,* 21 SCX fractions). This fractionation strategy resulted in the quantification of > 2000 proteins from a single tissue sample (Supplemental Fig. 10). However, due to experimental design constraints, the remaining tissue samples were fractionated using SCX spin columns (*e.g.*, 7 SCX fractions). Although this strategy reduced the number of quantified proteins by ~ threefold, meaningful glycoprotein expression data were obtained from these prostate tissue samples (Supplemental Fig. 10, Supplemental Excel file 3).

To identify glycoprotein signatures that distinguish between to BPH, PCa, and mPCa, we compared glycoprotein expression across all tissue samples. Supervised hierarchical clustering uncovered sets of glycoproteins that are upregulated in BPH relative to localized PCa and mPCa and, conversely, signatures of sets that are upregulated in localized PCa and mPCa relative to BPH (Fig. 3C). For example, lymphocyte-derived immunoglobulins were more abundant in BPH samples than PCa and mPCa samples (Fig. 3C, lower image), consistent with the notion that inflammation underlies the initiation and progression of BPH^47,48^. In contrast, glycoproteins involved in proteasome function (*e.g.* PSMD4)^49,50^, *N*-linked glycosylation (*e.g.,* ENTPD5)^51–54^, glycan metabolism (*e.g.,* NAGA)^55^, and receptor trafficking (*e.g.,* LAMP1)^56^ were upregulated primarily in localized PCa and mPCa (Fig. 3C, upper image). These results show that our glycoproteomic profiling experiments have the power to detect differences in glycoprotein expression in clinical prostate tissue specimens.

**Figure 4:**
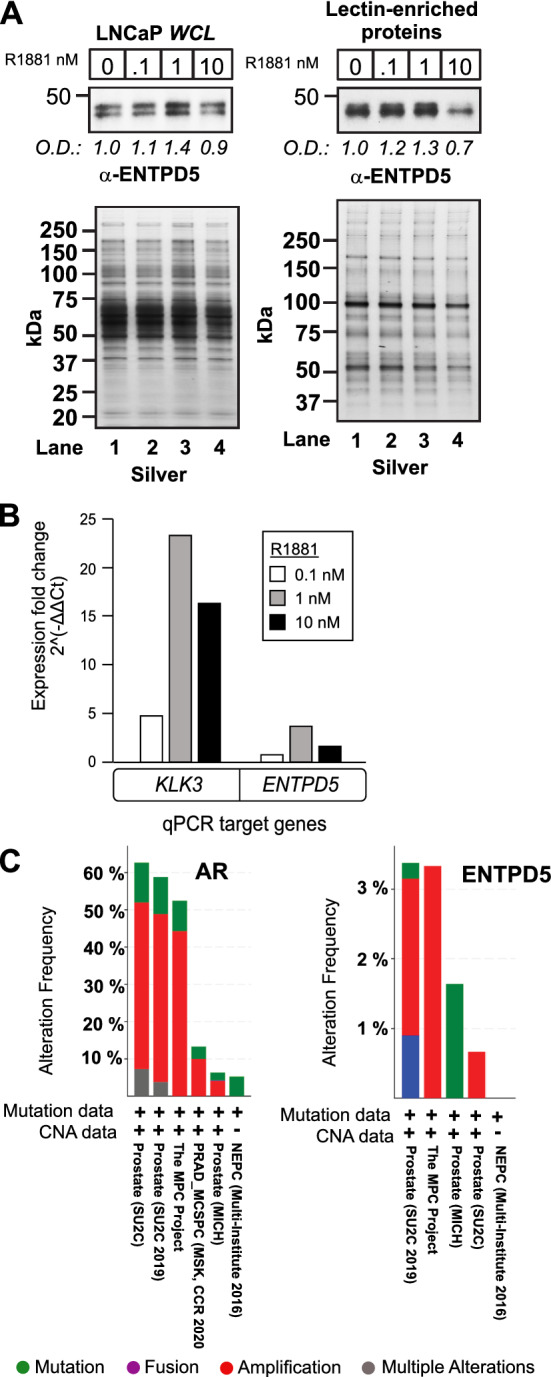
ENTPD5 expression is regulated by androgens in LNCaP cells. (**A**) (Upper panel) Dose-dependent ENTPD5 protein expression in androgen-treated LNCaP cells, in whole-cell lysates (WCLs) and lectin-enriched membrane protein extracts, as assessed by western blotting. (Lower panel) Silver-staining to verify equivalent protein loading for *WCL* and lectin-enriched protein samples. Western blot signals were normalized to the silver staining and quantified using the Image J software program^59^ (**B**) *ENTPD5* expression in androgen-treated LNCaP cells. *KLK3* (*PSA)* is a canonical androgen-regulated gene in androgen-responsive prostate tumor cells and served as a positive control. Mean and values and standard deviations were derived from three technical replicate wells (Results from a single biological experiment are shown; QPCR results from 2nd and 3rd independent biological experiments are shown in Supplemental Fig. 11). (**C**) OncoPrint genomic alterations of AR and ENTPD5 in advanced human prostate cancers. The three studies contained 325 samples obtained from 290 patients.

Given that androgen-regulated gene-expression programs are frequently dysregulated in early-stage PCa^3,57^, we decided to use androgen-regulated glycoproteins in LNCaP cells as biomarkers to guide the discovery of candidate androgen-regulated glycoproteins in clinical prostate tissue samples (Fig. 3D). Supervised hierarchical clustering of glycoproteins from LNCaP, as well as BPH, localized PCa, and mPCa samples, uncovered many glycoprotein clusters between samples (Fig. 3D). We focused on LNCaP glycoproteins whose levels changed in response to androgens (*i.e.,* androgen-mediated increases or decreases) and that were upregulated in localized PCa and/or mPCa samples. One such protein was ENTPD5. Its overall levels were regulated by androgens in LNCaP cells and were higher in both localized PCa and mPCa samples than in BPH samples (Fig. 3D). Moreover, levels of glycosylated ENTPD5 were consistently higher in PCa tissues than in NAT (Fig. 3D). Notably, they were highest in cells treated with 1 nM androgen (Fig. 4B), the concentration of androgen that induced maximal proliferation of LNCaP cells, and undetectable in cells treated with 10 nM androgen, the dose that antagonized the proliferation of LNCaP cells (Supplemental Fig. 1). Cluster analysis revealed that levels of glycosylated β-catenin (CTNNB1), which is also regulated by androgens in LNCaP cells^58^, were higher in localized PCa and mPCa samples than BPH samples (Fig. 3D). These findings support the validity of identifying candidate androgen-regulated glycoproteins in LNCaP prostate-tumor cells based on their overexpression in cancerous prostate tissue.

ENTPD5 promotes the proliferation of cancer cells and is frequently overexpressed in cancerous tissues^51,60^. To verify that androgens regulate the expression of ENTPD5 in prostate-tumor cells, LNCaP cells in *AD* growth medium were challenged with various concentrations of androgen (24 h), generated whole-cell lysates, and performed Western blot analysis of ENTPD5. Overall protein levels increased up to 1 nM androgen, but were lower at 10 nM androgen (Fig. 4A, left panel). We also probed for glycosylated ENTPD5, using lectin-enriched microsomes. Exposure to up to 1 nM androgen led to increased glycosylation of ENTPD5, whereas exposure to 10 nM androgen caused a noticeable reduction in glycosylation (Fig. 4A, right panel). At the 10 nM dose, the reduction in glycosylated ENTPD5 was greater than the reduction of total ENTPD5, showing that this modification is suppressed by SPA in LNCaP cells. Overall, the results verified that ENTPD5 is an androgen-regulated glycoprotein in LNCaP cells.

These findings prompted us to determine whether ENTPD5 expression is also transcriptionally regulated by androgens, which would support its designation as an *ARG* in LNCaP cells. Androgen exposure caused a dose-dependent increase in ENTPD5 gene-transcripts at up to the 1 nM concentration, and a slight reduction in ENTPD5 gene expression was observed at 10 nM androgen by qPCR analyses (Fig. 4B, Supplemental Fig. 11). Importantly, the *KLK3* gene, which is a direct target of AR-dependent transcription and a canonical *ARG* in androgen-responsive prostate cancers^61–63^, showed the same biphasic transcriptional response to androgen (Fig. 4B). This result prompted us to search for genomic AR binding sites in *ENTPD5*; their presence would provide further evidence that it is a downstream target gene of AR. Thus, genomic AR binding sites identified in a previous chromatin immunoprecipitation sequencing (ChIP-Seq) study were re-examined in androgen (*i.e.,* R1881)-treated LNCaP cells^4^. Notably, ChIP-seq signals at *ENTPD5* localized to introns 2 (chr14:74,482,139–74,482,531, 375 base pairs) and 6 (chr14:74,458,755–74,459,147, 125 base pairs) (Supplemental Fig. 12A)^4^. Although the MEME motif program failed to identify canonical androgen response element (ARE) sequences (*e.g.*, AGAACANNNTGTTCT)^64^ in either DNA segment, a 15-bp consensus motif, CCASBANNYCCAGCY, which was the longest and most abundant (7 copies), was detected in both introns (Supplemental Fig. 12A). The Tomtom motif comparison tool showed that this sequence has strong homology to consensus motifs in ETS-family transcription factors ELK4 and ETS1, as well as to motifs in the ladybird homeobox 2 (LBX2) transcription factor (Supplemental Fig. 13B). Notably, AR and ETS1 physically interact and coregulate a subset of *ARGs* in LNCaP cells^65^. Interestingly, evaluation of clinical prostate cancer datasets at the cBioPortal for Cancer Genomics showed that *ENTPD5* is infrequently amplified (~ 3%) in a subset of CRPCs (Fig. 4C)^66,67^, which suggest ENTPD5 overexpression may have some role in the progression of late-stage PCa. Our findings show that *ENTPD5* is an *ARG* that is transcriptionally regulated, either directly or indirectly, through complex interactions between AR and auxiliary transcription factors in LNCaP cells.

## Discussion

The recent success of bipolar androgen therapy (BAT), which restores ADT sensitivity to a subset of CRPCs, has spurred greater clinical interest in the treatment of CRPC with SPA^68–71^. Additionally, a recent study showed that SPA suppressed proliferation in patient-derived xenografts of CRPC^72^. SPA-mediated inhibition of the proliferation of PCa cells is caused by AR-dependent transcriptional mechanisms involving the repression of the transcription factors that underlie cell growth (*e.g.*, c-MYC, E2F)^35^, the upregulation of cell-cycle inhibitors (*e.g.*, p27, p21, Skp2)^73,74^, the induction of terminal differentiation (*e.g.*, APRIN, PLZF)^75–80^, the repression of DNA replication genes^81^, the repression of AR and AR variants^37,81,82^, and activation of cell senescence through the repression of E2F-regulated genes^36,39^. Transcription-independent mechanisms underlying SPA-mediated inhibition of proliferation by PCa cells include activation of the DNA double-strand break damage response (DDR)^83^ and the inhibition of DNA licensing by AR stabilization at pre-replication complexes during M phase^34,84^. In this glycoproteomics study of LNCaP cells, we found that SPA coordinates the expression of glycoproteins involved in the biological pathways SRP-dependent protein cotranslational targeting to membrane, Negative regulation of ubiquitin protein ligase activity, and Positive regulation of DNA damage response. How glycosylation status is coupled to SPA-mediated inhibition of PCa-cell proliferation will require further exploration. Given the enrichment of ribosomal proteins among the SPA-enriched glycoprotein networks, and the fact that ribosomal proteins are targeted for *O*-GlcNAcylation during protein translation and stress-granule (SG) formation^85–87^, we speculate that they are *O*-GlcNAcylated. Given that *O*-GlcNAcylation regulates translation initiation, stabilizes nascent polypeptide chains during cotranslation, and triggers SG disassembly for the translation of stress mRNAs^88–90^, it is possible that SPA elicits a stress response in PCa cells that antagonizes proliferation through the formation of stalled translation preinitiation complexes and SGs^91^.

Previous studies showed that SPA induces dsDNA breaks in PCa cells^70,83^, suppresses the gene expression proteins that drive DDR and homologous recombination in CRPC patient-derived xenografts (PDXs)^72^, and mediates an extreme response to BAT in a patient who harbors a germline missense mutation in the serine/threonine protein kinase-encoding *ATM* gene and a frameshift mutation in the breast cancer gene BRCA2^92^. As reported in the current study, SPA enriches for the biological pathway that represents Positive regulation of DNA damage response in LNCaP cells, providing further experimental support for previously reported physical and functional interactions among AR, topoisomerase II (TOP2), and the DNA repair machinery in human PCa^83^. Our findings justify further exploration of whether DNA damage response proteins are *O*-GlcNAcylated, and whether such O-GlcNAcylation is functionally coupled to SPA-mediated inhibition of PCa proliferation in humans.

*O*-GlcNAcylation can antagonize phosphorylation-dependent ubiquitination to modulate the stability of transcription factors and protein kinases in a variety of cellular models^93,94^. *O*-GlcNAcylation of the ubiquitin–proteasome system (UPS) antagonizes proteasome activity through the *O*-GlcNAcylation of the RPT2 ATPase, a component of the 19S proteasome^94,95^. Our discovery that SPA-treated LNCaP cells were enriched for the biological pathway Negative regulation of ubiquitin protein ligase activity suggests that *O*-GlcNAcylation might potentiate ubiquitin-mediated protein degradation in response to SPA. This is based on previous work showing that global ubiquitination decreases upon an increase in *O*-GlcNAc or upon knockdown of *O*-linked N-acetylglucosamine transferase (OGT)^96^. For example, *O*-GlcNAcylation promotes the stability of nascent polypeptides during protein cotranslation because OGT inhibition facilitates the poly-ubiquitination and premature degradation of proteins that are cotranslated proteins by the UPS^89^. Our findings justify further scientific exploration of whether a functional relationship exists between *O*-GlcNAcylation, the UPS, and SPA-mediated inhibition of PCa cell proliferation.

The elucidation of androgen-regulated gene-expression programs that control the proliferation of PCa cells has been the focus of many research studies seeking to shed light on how defects in the expression of *ARGs* contribute to the development and/or progression of human PCa^27,28,97–100^. We have expanded beyond this genomic perspective and shown, for the first time, how androgens elicit dose-dependent changes in glycoprotein expression in LNCaP PCa cells. Androgens are known to transcriptionally regulate protein glycosylation pathways in PCa cells, directly through AR-dependent mechanisms^101^. Twenty-five *ARGs* in the protein glycosylation pathway have been shown to encode enzymes that act at different steps of the hexamine biosynthesis pathway (HBP)^102^, *N*- and *O*-glycan biosynthesis, and chondroitin sulfate (CS) synthesis^103^. Our glycoproteomic profiling experiment shows that the enzyme ENTPD5, which promotes *N*-glycosylation and ER protein folding, and also contributes to the Warburg effect in PCa cells^51^, is an androgen-regulated glycoprotein in LNCaP cells. Moreover, we present experimental data showing that ENTPD5 expression is regulated transcriptionally (AR-dependent) and potentially post-transcriptionally (glycosylation) by androgens in this cell type. Of note, the overexpression of ENTPD5 correlates with AKT activation in primary tumors, and ENTPD5 expression is required for the glycosylation of growth factor receptors (*e.g.*, EGFR, Her2, IGF-IRβ) in PCa cells^51^. In LNCaP cells, levels of glycosylated ENTPD5 were reduced following SPA treatment, suggesting that this modification might be functionally coupled to SPA-mediated inhibition of proliferation. Thus, it is possible that a reduction in glycosylated ENTPD5 leads to a reduction in glycosylated growth factor receptors and to a subsequent reduction in growth factor-mediated proliferation signals in PCa cells. Experiments to clearly establish a functional relationship between ENTPD5 glycosylation and SPA-mediated inhibition of proliferation in PCa cells requires further scientific inquiry.

Although this glycoproteomics study uncovered novel androgen-regulated proteins in both PCa cells and clinical prostate tissues, it has several experimental limitations. Firstly, it does not define site-level mass spectrophotometric determination of *N*-linked and *O*-linked residues. The LWAC method enriched for detergent-solubilized microsomal glycoproteins in LNCaP cells and clinical tissue under non-denaturing conditions. Thus, the glycoproteomic output might have been contaminated with non-glycosylated proteins that bound to bonafide *N*- and *O*-linked glycosylated proteins through piggyback interactions. The incorporation of methods using solid phase extraction of glycopeptides (SPEG) followed by either the enzymatic release of *N*-linked glycopeptides^104,105^ or the chemical release or *O*-linked glycopeptides^106,107^ would facilitate site-level mass spectrophotometric determination of *N*-linked and *O*-linked residues in lectin-affinity purified glycoprotein samples respectively. Secondly, the accuracy of resection of tissue sections was controlled by a uni-core cutting tool. The use of laser capture microdissection (LCM) methods would improve the accuracy of this aspect of the study. For example, our comparative glycoprotein expression analyses of BPH and PCa samples was limited because BPH samples are heterogeneous in cellular composition, and the samples likely contain smooth muscle cells, fibroblasts, and both secretory and basal epithelial cells. LCM methods would afford greater accuracy in the resection of epithelial cells from these heterogenous samples so that true expression differences in androgen-regulated glycoproteins between epithelial cell-types between BPH and PCa samples could be determined. Thirdly, the number of available clinical samples used for glycoprofiling limited the power of the study. Increasing the number of samples would increase the power to detect candidate biomarkers in diseased tissue samples. Notwithstanding these experimental limitations, we anticipate that the glycoproteomic findings presented here will provide new insights into how androgens regulate glycoprotein networks in PCa cells. In addition, this subpopulation of proteins might represent a rich resource of candidate biomarkers of cellular diseases that affect the prostate gland.

## Methods

### Materials

LNCaP cells were from American Type Culture Collection; Dulbecco’s Phosphate Buffered Solution, phenol red-deficient RPMI 1640 media, 10X Glutamax, and 10X penicillin and streptomycin were from Invitrogen; normal and charcoal stripped fetal bovine serum were from Hyclone Laboratories (Logan, UT) (Invitrogen); protease inhibitor cocktail tablets and dithiothreitol (DTT) were from Thermo Scientific Pierce; Wheat germ agglutinin and Concanavalin A-agarose beads were from Vector Laboratories Inc. (Burlingame, CA). Tissue biopsy punch tools, sugars, solvents (*i.e.*, non-organic and organic), and all other chemicals were from Sigma-Aldrich. Western blot antibody reagents included: a rabbit polyclonal antibody to prostate-specific antigen (PSA) from DAKO (catalog #A0562)(Carpinteria, CA), a mouse monoclonal to Hemagglutinin A (HA) (catalog #2367), and rabbit polyclonal antibodies to DNA-dependent protein kinase catalytic subunit (PRKDC)(catalog #4602) and glyceraldehyde 3-phosphate dehydrogenase (GAPDH)(catalog #2118) were from CST (Danvers, MA), mouse monoclonal antibodies to sodium/potassium (Na/K) ATPase (ATP1B1)(catalog #sc-21712) and AR (catalog # AR441) from Santa Cruz Biotechnology (Santa Cruz, CA), a mouse monoclonal antibody to clathrin heavy chain (CLTC)(catalog #610499) from BD Biosciences (San Jose, CA), a mouse monoclonal antibody to ectonucleoside triphosphate diphosphohydrolase 5 (ENTPD5)(catalog # 743512) from R&D Systems (Minneapolis, MN). The compound methyltrienolone (R1881) was purchased from Perkin Elmer (Waltham, MA), and Enzalutamide (catalog #S1250) was purchased from Selleckchem (Houston, TX). Sequence-grade trypsin (catalog #V5113) was purchased from Promega (Madison, WI). The bicinchoninic acid (BCA) protein assay kit (catalog #23228), Slide-A-Lyzer dialysis cassettes (catalog #’s 66373, 66380), Dulbecco’s Phosphate Buffered Solution (PBS)(catalog #14040141), SuperScript® III First-Strand Synthesis kit (catalog #18080400), Oligofectamine (catalog # 12252011), Lipofectamine 2000 Transfection Reagent (catalog # 11668027), and 4–12% Bis–Tris gels (catalog #NP0336BOX), and the CyQUANT Cell Proliferation Assay Kit (catalog # C7096) were from ThermoFisher Scientific (Waltham, MA ). C18 (catalog #SEM SS18V) and strong cation exchange (SCX) macrospin (catalog #SMM HIL-SCX.25) and microspin (catalog #SEM HIL-SCX) columns were from The NEST Group Inc (Southborough, MA). Sep-Pak tC18 cartridges (1 cc-100 mg) (catalog WAT036820) were purchased from Waters (Milford, MA). Trypsin-digested iodoacetic acid alkylated bovine serum albumin (BSA)(catalog #PTD00001-15) was from (Michrom Bioresources (Auburn, CA). The RNeasy Midi Kit (catalog #75142), GAPDH (catalog #PPH00150F), KLK3 (catalog #PPH01002B), and ENTPD5 (catalog # PPH12102B) qPCR primers were from Qiagen (Germantown, MD). The SYBR Green PCR Master Mix (catalog #4344463) was from Applied Biosystems (Foster City, CA), and 48-well tissue culture dishes (catalog #353230) were from BD Bioscience (San Jose, CA). The In-Fusion® HD EcoDry™ Cloning Plus kit (catalog #638912) was from Takara Bio USA (Mountain View, CA).

### Clinical samples

37 fresh-frozen prostate tissue samples, representing 29 human subjects, were accrued from academic and commercial sources. The first set of tissue samples was derived from four human subjects and consisted of paired samples of tumor tissue (TT) and normal adjacent tissue (NAT) accrued by the University of Iowa Tissue Core (Iowa City, IA) (Supplemental Table III). The second set of tissue samples was obtained from fifteen subjects and consisted of five paired TT and NAT samples, and 10 BPH samples accrued from Proteogenex (Culver City, CA). The third set of tissue samples was from 10 subjects and composed of TT accrued from Bioserve Biotechnologies LTD (Beltsville, MD). Aside from 2 of the TT samples, all others had a Gleason score of ≥ 7. This included 9 stage-II tumor samples, 8 stage-III tumor samples, and 3 stage-IV samples (Supplemental Table II and III). All tissue sample hematoxylin and eosin (H&E) stained slides were evaluated by a certified clinical pathologist, and regions of TT and NAT were denoted on H&E slide to guide the resection of frozen tissue samples. For all BPH samples, the entire tissue sample was processed for glycoprotein extraction and processing for mass spectrometry analysis. Ethical approval and consent to participate: Tumor samples were obtained under informed consent after approval by the University of Iowa Institutional Review Board: IRB#200907702 and #201103721 protocol. All data collection, processing, and consenting process were executed after approval by the IRB at the University of Iowa. Also, all methods were performed in accordance with the relevant guidelines and regulations of the IRB.

### Tissue protein extraction

A 5 mm Uni-core cutting tool was used to resect TT and NAT samples (Fig. 3, Supplemental Fig. 7). Cored as well as BPH samples were suspended in ice-cold PBS (containing CaCl_2_ and MgCl_2_), briefly vortexed, and centrifuged at 4 °C at 557 × g for 5 min to remove non-tissue contaminants, including optimum cutting temperature (OCT) medium, blood, and cellular debris. Each sample was resuspended in membrane extraction buffer (MEB) (20 mM Tris, 150 mM NaCl, 0.1 mM CaCl_2_, 0.1 mM MnCl_2_, 1 × Halt Protease Inhibitor Complex, 10 mM DTT, 5 mg/ml Digitonin) and rotated end-over-end overnight at 4 °C. The samples were centrifuged at 1200 × g for 8 min at 4 °C and the collected supernatants were centrifuged for another 1 h at 100,000 × g at 4 °C. The supernatants were collected and quantified by silver-stained gel analysis; detergent-solubilized LNCaP whole-cell lysates were used as references for standard for protein quantification (Supplemental Figs. 8 and 9).

### Enrichment of glycosylated microsomal proteins from LNCaP prostate-tumor cells

#### Large-scale experiment

LNCaP prostate-tumor cells grown in 500 cm tissue culture plates for 72 h in androgen-depleted (*AD*) growth medium (phenol-red free RPMI 1640 + 10% charcoal-stripped FBS) were exposed to vehicle (EtOH) or synthetic androgen R1881 at 0.1, 1, and 10 nM for 24 h. Cells were washed twice with ice-cold PBS, scraped from the plates, and centrifuged for 5 min at 1800 rpm at 4 °C. The supernatants were decanted, and cell pellets were resuspended into hypotonic lysis buffer (HLB) (10 mM Hepes, 1.5 mM MgCl_2_, 10 mM KCl, pH 7.9, 5 mM DTT-Sigma, and 1 × Halt Protease Inhibitor Complex). The hypotonic samples were incubated on ice for 10 min and subjected to nitrogen cavitation (*i.e.,* 100 psi) for 5 min. Nitrogen-cavitated samples were centrifuged at 600 × g for 20 min at 4 °C to pellet out intact nuclei and unbroken cells. The supernatants were collected and centrifuged at 100,000 × g for 3 h at 4 °C. The supernatant (*i.e.,* cytosolic protein fraction) was removed and the crude microsomal pellet, which contained intact organelles and membrane microsomes, was solubilized in MEB. The samples were rotated end-over-end for 16 h at 4 °C, and then subjected to 100,000 × g centrifugation for 1 h at 4 °C to remove detergent-insoluble particulate matter. Collected supernatants were quantified by silver-stained gel analysis, using detergent-solubilized LNCaP whole-cell lysates as a reference standard for protein quantification (Supplemental Fig. 8). The digitonin-solubilized samples (*i.e.,* 10 mg protein) were incubated and rotated end-over-end overnight with a mixture of wheat germ agglutinin (WGA) and concanavalin A (ConA) agarose beads (Vector Laboratories Inc., Burlingame, CA) in MEB at 4 °C. Non-specific, non-glycosylated proteins were removed by three consecutive washes with MEB. Glycosylated proteins were competitively eluted by incubating each sample with MEB supplemented with 500 mM N-acetyl-D-glucosamine, 200 mM α-methyl mannose, 200 mM α-methyl glucose, and 200 mM α-D-mannose for 30 min at 4 °C. Supernatants were collected and loaded into 10 kDa cutoff dialysis cassettes and subjected to overnight dialysis in urea buffer (UB) (8 M Urea, 50 mM Tris, and 100 mM β-mercaptoethanol, pH 8.5). Collected supernatants were quantified by silver-stained gel analysis, using detergent-solubilized LNCaP whole-cell lysates as a reference standard for protein quantification (Supplemental Fig. 9).

#### Small-scale experiment

LNCaP cells were treated and processed for glycoprotein enrichment exactly as described in the large-scale experiment above, except that LNCaP cells were grown in 10 cm^2^ tissue culture dishes for this experiment.

### Strong-cation exchange peptide fractionation

#### LNCaP microsomal glycoproteins

Lectin-affinity enriched protein extracts derived from vehicle (EtOH) and androgen-treated (*i.e.,* 0.1, 1.0, and 10 nM R1881) LNCaP cells were trypsin-digested and peptides were fractionated by strong-cation exchange (SCX) chromatography prior to directed mass spectrometry (dMS) analyses. For each experimental condition (*i.e.,* EtOH, 0.1, 1.0, and 10 nM R1881), 350 µg of protein was reduced with DTT (10 mM) for 1 h at 37 °C and alkylated with iodoacetamide (55 mM) for 1 h at room temperature in the dark. Each sample was diluted into Tris buffer (50 mM, 0.5 M Urea, pH 8.5) and trypsin-digested overnight at 37 °C (*i.e.,* 1:50 trypsin: protein ratio). Samples were then acidified (pH ~ 3) with phosphoric acid, and acetonitrile (ACN) was added to achieve a final concentration of 1%. Trypsin-digested iodoacetic acid alkylated BSA was introduced into each sample at a 1:75 protein ratio and subsequently desalted on Sep-Pak tC18 cartridges at a flow-rate of 1 ml/minute in buffer A (1% ACN, 1% TFA). The peptide samples were speedvac evaporated and loaded onto a 2.1 mm × 20 cm Polysulfoethyl A column (PolyLC Inc., Columbia, MD) in buffer A (5 mM KH_2_PO_4_, 25% acetonitrile, pH 2.8). The peptides were eluted with an increasing concentration of buffer B (*i.e.* flow-rate of 250 µl/min, 5 mM KH_2_PO_4_, 350 mM KCl, 25% acetonitrile, pH 2.8) from 0–59% buffer B for 30-min on the Agilent 1200 HPLC system. Collected samples were desalted over C18 spin columns and analyzed by dMS.

#### Clinical tissue samples

Digitonin-extracted proteins from TT and NAT (*i.e.,* R90197381, R93776568, R0318823, and R42521246), underwent lectin-affinity chromatography, trypsin-digestion, and SCX–HPLC; the method was identical to that described above for glycosylated protein samples derived from LNCaP microsomes except that in this case 100 µg of trypsin-digested protein was fractionated by SCX–HPLC. A total of 22, 21, 21, and 12 SCX fractions were collected and processed for tissue samples R03188233, R42521246, R93776568, and R909197381, respectively. All remaining tissue samples were SCX fractionated on silica-based macrospin (*i.e.,* 50–500 µg capacity) or microspin columns (*i.e.,* 10–100 µg capacity). Samples were resuspended in buffer A. The columns were conditioned and primed according to the manufacturer’s protocol. Sample-loaded spin columns were SCX fractionated with concentrated salt buffer (buffer C) (5 mM KH_2_PO_4_, 25% ACN, 300 mM KCl, pH 2.8). Peptides were eluted with an increasing gradient of KCl bumps created by mixing different proportions of buffer A and buffer C. Tissue samples from Proteogenex were SCX fractionated with KCl bumps at 20 mM, 25 mM, 40 mM, 50 mM, 60 mM, and 180 mM. Tissue samples from Bioserve Biotechnologies were SCX fractionated with KCl bumps at 15 mM, 20 mM, 25 mM, 40 mM, 50 mM, 60 mM, 180 mM. Samples were desalted on microspin C18 columns prior to dMS analyses, as detailed below.

### qPCR experiments

LNCaP cells grown for 72 h in *AD* growth medium were exposed to vehicle (*i.e.*, EtOH) or the synthetic androgen R1881 at 0.1, 1, and 10 nM for 24 h. Total RNA was extracted from vehicle and androgen-treated cells using the RNeasy Midi Kit. First-strand cDNA synthesis was performed with the SuperScript® III First-Strand Synthesis kit, and real-time quantitative PCR was performed with the SYBR Green PCR Master Mix, using qPCR primers directed to GADPH, AR, and ENTPD5. Normalized Ct values in experimentally-treated samples at 0.1, 1, and 10 nM R1881 were determined based upon GAPDH, KLK3, and ENTPD5 gene expression values in vehicle-treated samples using the Ct method.

### Liquid chromatography mass spectrometry

Desalted, tryptic peptide samples were dissolved in mass spectrometry loading buffer (1% acetic acid, 1% acetonitrile) and analyzed by nanoliquid chromatography-tandem mass spectrometry using an Agilent 6520 Accurate-Mass Quadropole Time-of-Flight mass spectrometer interfaced with an HPLC Chip Cube. The samples were loaded onto an Ultra High Capacity Chip (500 nL enrichment column, 75 μm × 150 mm analytical column). LC–MS/MS analysis was performed using a 120-min gradient ranging from 1.5 to 32% buffer C (100% acetonitrile, 0.8% acetic acid). Full MS (MS1) data was acquired using a mass range of 400–1250 m/z and acquisition rate of 1 spectra/second. From these data, an ion preferred list was generated with Agilent MassHunter Qualitative Software, with the settings of 400–1250 m/z, 2 + and 3 + charge states, and spectra with 2 or more ions. Directed mass spectrometry (dMS) was performed with the following settings: a maximum of 10 ions per cycle, a narrow isolation width (~ 1.3 atomic mass units), precursor masses dynamically excluded for 30 s after 8 MS/MS in a 30-s time window and use of the preferred ion list. Mass spectrometry capillary voltage and capillary temperature settings were set to 1800 V and 330 °C, respectively. The infused reference mass of 1221.9906 was used to correct precursor m/z masses each LC–MS/MS experiment.

### Mass spectrum data analysis

#### Extraction

Spectral extraction and merging were performed using Agilent’s Technologies Spectrum Mill extraction software. A static modification of carbamidomethylation (C = 57) was used for pre-search filtering, and mass (MH +) range of 50 to 2000 mass–charge ratio (m/z) and scan time range of 10 to 130 min were used for MS/MS spectral feature filtering. Retention time tolerance was 60 s, m/z tolerance was 0.015 m/z, auto charge was set to default, MS Noise Threshold was set to 5, and Spectral Similarity & RT & m/z were used as general MS/MS merging constraints.

#### MS search

The tissue samples were searched against the 14 February 2013 release of the UniProt human database with a parent mass tolerance of 25 ppm and fragment mass tolerance of 200 ppm, using the Agilent Technologies Spectrum Mill Software (Ver B.04.00.127). The search modifications included a double trypsin miss cleavage, a static carbamidomethylation on cysteine residues (C = 57.02146 AMU), differential modifications for oxidized methionine (M = 15.9949 AMU), and phosphorylated oxidized methionine, serine, tyrosine, and threonine (STY = 79.9663 AMU), and N-acetylglucosamine oxidized methionine, serine, tyrosine, and asparagine. The masses for the modifications phosphorylation, carbamidomethylation and oxidation are 79.9663, 57.02146, and 15.9949 AMU, respectively.

#### Post search

The data were filtered based upon the Spectrum Mill Forward-Reverse Score threshold of 1.2, Rank 1–2 score threshold of 2, Score threshold of 3 and %SPI threshold of 30. The spectral intensity of the identified protein was normalized to the ratio of the tumor over normal experiments of identified bovine serum albumin carboxymethylated cysteine (C = 58.0055 AMU) peptides total intensities for equal sample loading.

### Bioinformatic analyses

#### *Cluster analyses of lectin-enriched proteome-Fig. **2**A*

Enriched network pathway analyses were performed using the WEB-based Gene SeT AnaLysis Toolkit “WebGestalt”. Spectrum Mill identified proteins in vehicle and androgen-treated groups (*i.e.*, 0, 0.1, 1.0, and 10 nM R1881) were uploaded individually into the WebGestalt program. UniProt IDs were subjected to enriched pathway analyses with KEGG Pathway as the function database and default parameters^108^. The enriched network pathways and computed *p*-values < 0.05 were calculated as −log(*p*-value) and transformed as a heatmap in the TreeView software.

#### Supervised clustering of lectin-enriched proteome between LNCaP cells and clinical tissue samples

GenePattern^109^ supervised clustering was performed on protein intensity values ranging from 1.03 × 10^4^ to 2.77 × 10^9^. The values were log transformed and uploaded into GenePattern for hierarchical clustering. The columns (samples) were fixed and the rows (identified proteins) were clustered using Pearson correlation as the clustering criterion with pairwise complete-linkage as the hierarchical clustering method. The exported clustered results were visualized in the TreeView software with the maximum value capped to 5 on the scale bar to easily visualize changes in protein expression across samples.

## Supplementary Information

Supplementary Information 1.

Supplementary Information 2.

Supplementary Information 3.

Supplementary Information 4.
