# Supplementary material for: Discovery proteomics defines androgen-regulated glycoprotein networks in prostate cancer cells, as well as putative biomarkers of prostatic diseases
Source: Sci Rep. 2021 Nov 15;11:22208. doi: 10.1038/s41598-021-01554-2 (PMC8592995; doi:10.1038/s41598-021-01554-2)
Supplement: Supplementary file 1 — Supplementary Information 1. [file 41598_2021_1554_MOESM1_ESM.pdf]

Supplementary Figures and Tables

Table of Contents ……………………………………………………………………………Page #

Supplementary Figure 1A-1B… Androgen-mediated proliferation is dose-dependent in LNCaP prostate-tumor cells 2

Supplementary Figure 2...Full western blot panels 3

Supplementary Figure 3... Network visualization of enriched pathways in androgen-responsive glycoproteins in LNCaP cells……………………………………………………………………..4

Supplementary Figure 4…Theoretical androgen-regulated glycoprotein clusters……………….…5

Supplementary Figure 5…K-means androgen-regulated glycoprotein clusters………………………..6

Supplementary Figure 6…Androgen-regulated glycoprotein clusters associated with proliferation-inhibition in LNCaP cells………………………………………………………………………..7

Supplementary Figure 7…Light photomicrographs of hematoxylin and eosin-stained tissue samples…………………………………………………………………………………………8-11

Supplementary Figure 8 Silver-stained gels of detergent-extracted proteins from tissue samples..12

Supplementary Figure 9…Silver-stained gels of extracted glycoproteins from tissue samples after lectin chromatography………………………………………………………………………………13

Supplementary Figure 10…Protein identifications in clinical samples 14

Supplementary Figure 11…QPCR analyses of KLK3, GAPDH, and ENTPD5 in LNCaP cells 15

Supplementary Figure 12…Evaluation of transcription factor binding sites in *ENTPD5* introns #2 and #5…………………………………………………………………………………………………… 16

Supplementary Figure 13…Steps for clustering glycoproteins in LNCaP cells and tissue samples……………………………………………………………………………………… 17-23

Supplemental Figure Legends………………………………………………………………24-27

Supplementary Table I… Mass spectrometry statistics…………………………………………….28

Supplementary Table II…Clinical features of tissue samples………………………………………. 29

Supplementary Table III…Clinical features of tissue samples……………………………………….30

Supplementary Table IV Protein quantification of Normal Adjacent Tissue & Benign Prostate Hyperplasia……………………………………………………………………………………31-32

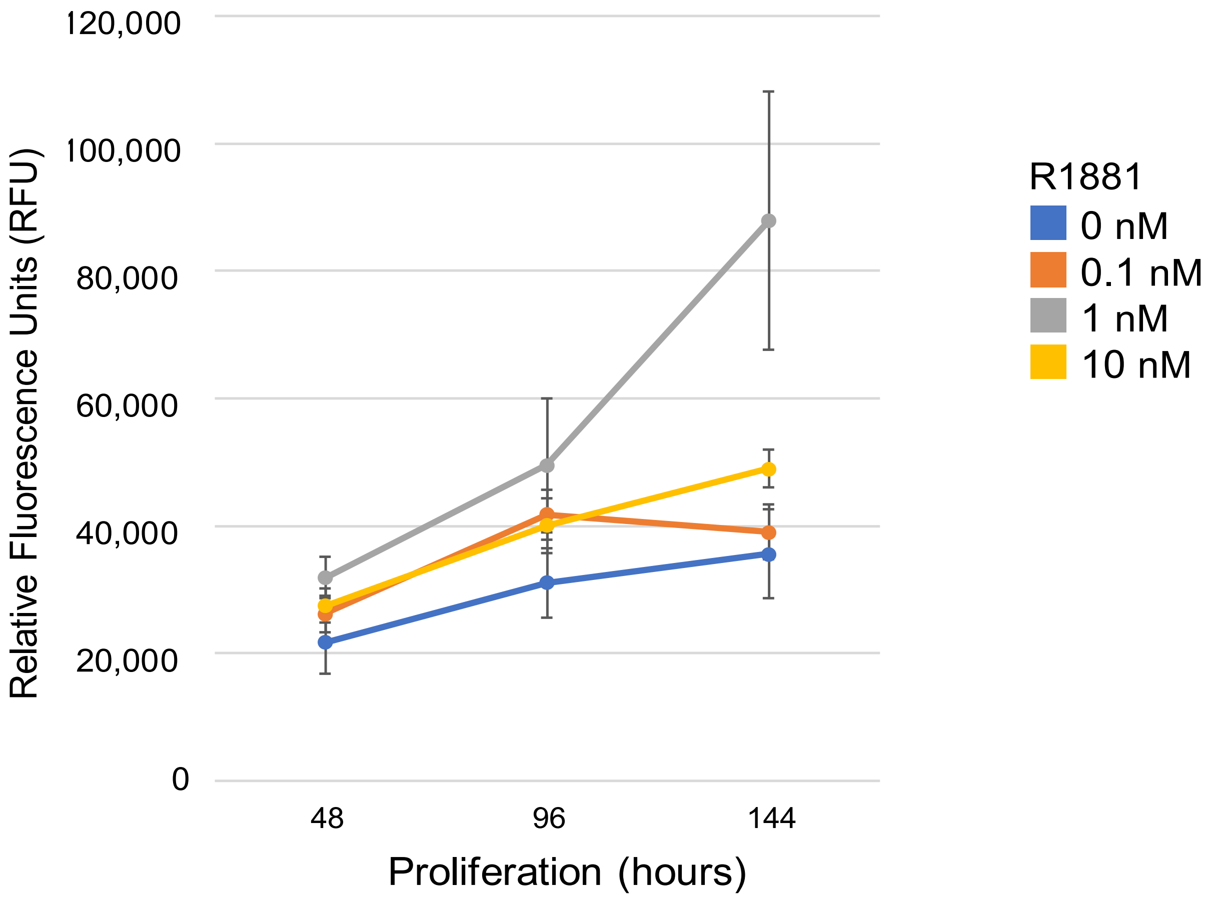

Supplemental Figure 1A. **Androgen-mediated proliferation is dose-dependent in LNCaP prostate-tumor cells.** LNCaP cells were seeded into a 96-well tissue culture plate containing androgen-depleted (*AD*) growth media (*i.e.*, phenol-red deficient RPMI1640 containing 10% charcoal-stripped serum) for 24hrs and challenged with vehicle (*i.e.*, EtOH; 0 nM R1881) and different concentrations of synthetic androgen R1881 (*i.e.*, 0.1, 1, or 10 nM). The relative fluorescence unit (RFU) of vehicle- and androgen-treated LNCaP cells was determined at 48 hrs (i.e. day 2), 96 hrs (*i.e.*, day 4), and 144 hrs (i.e. day 6) after the initial 24 hr seeding in *AD* growth medium with the CyQUANT Cell Proliferation Assay Kit. The RFU values for each experimental condition (12 wells per condition, *i.e.* 12 technical replicates) were graphed at 48, 96, and 144 hrs. The colored lines (*i.e.*, blue - 0 nM, orange - 0.1 nM, gray - 1 nM, and yellow - 10 nM) connect the mean values and standard deviation for each condition at 48, 96, and 144 hrs. The means were derived from twelve biological replicate wells per condition of a single biological experiment.

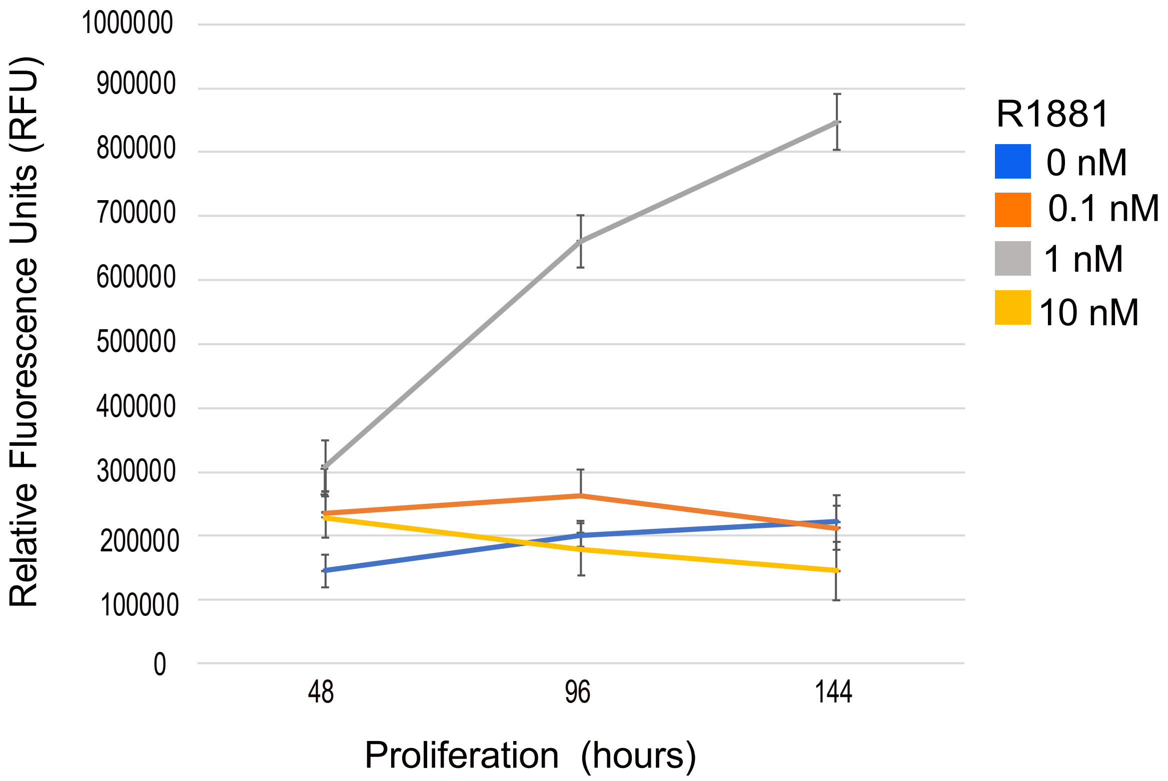

Supplemental Figure 1B. **Androgen-mediated proliferation is dose-dependent in LNCaP prostate-tumor cells.** LNCaP cells were seeded into a 96-well tissue culture plate containing androgen-depleted (*AD*) growth media (*i.e.*, phenol-red deficient RPMI1640 containing 10% charcoal-stripped serum) for 24hrs and challenged with vehicle (*i.e.*, EtOH; 0 nM R1881) and different concentrations of synthetic androgen R1881 (*i.e.*, 0.1, 1, or 10 nM). The relative fluorescence unit (RFU) of vehicle- and androgen-treated LNCaP cells was determined at 48 hrs (i.e. day 2), 96 hrs (*i.e.*, day 4), and 144 hrs (i.e. day 6) after the initial 24 hr seeding in *AD* growth medium with the CyQUANT Cell Proliferation Assay Kit. The RFU values for each experimental condition (12 wells per condition, *i.e.* 12 technical replicates) were graphed at 48, 96, and 144 hrs. The colored lines (*i.e.*, blue - 0 nM, orange - 0.1 nM, gray - 1 nM, and yellow - 10 nM) connect the mean values and standard deviation for each condition at 48, 96, and 144 hrs. The means were derived from twelve biological replicate wells per condition of an independent experiment.

Supplemental Fig. 1

Figure 1 Western Blots

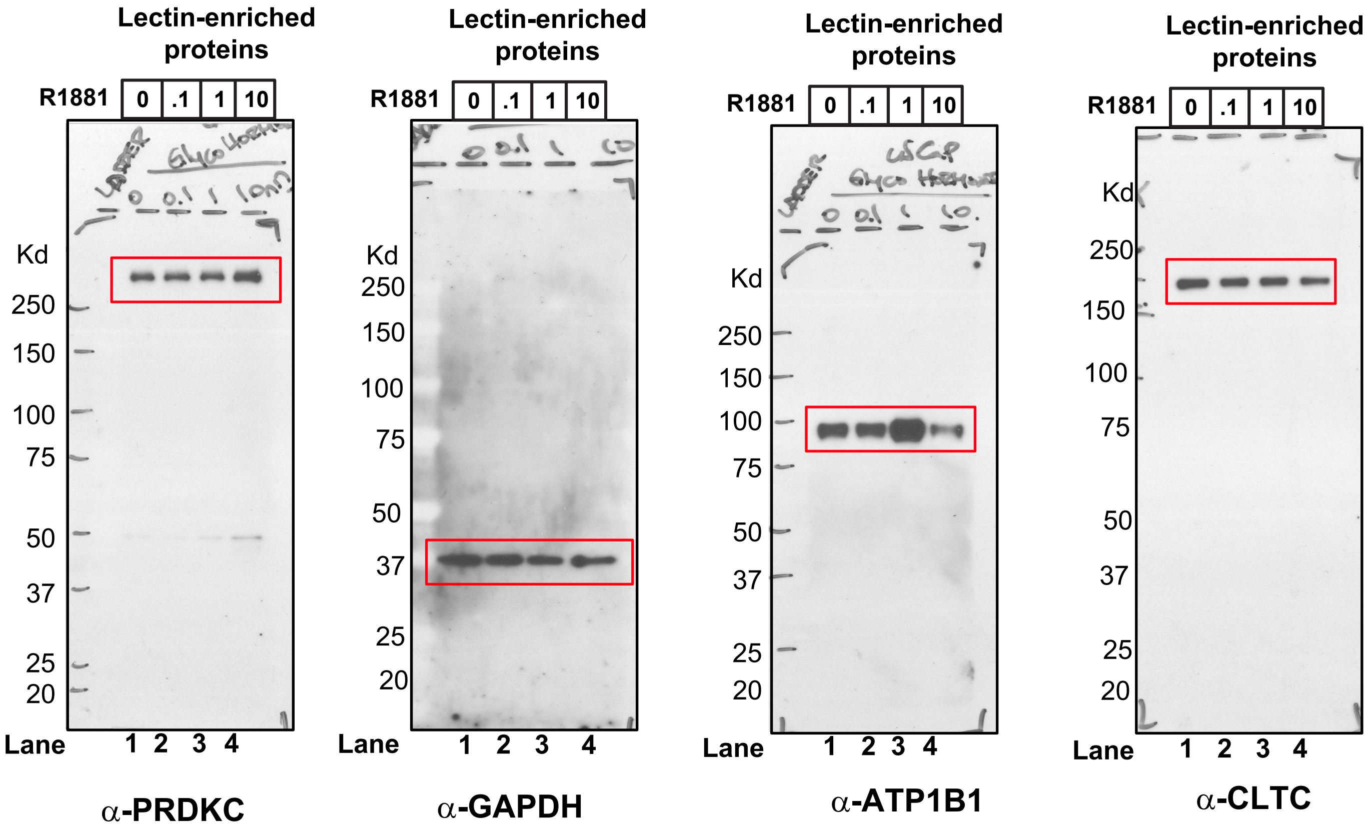

Figure 5 Western Blots

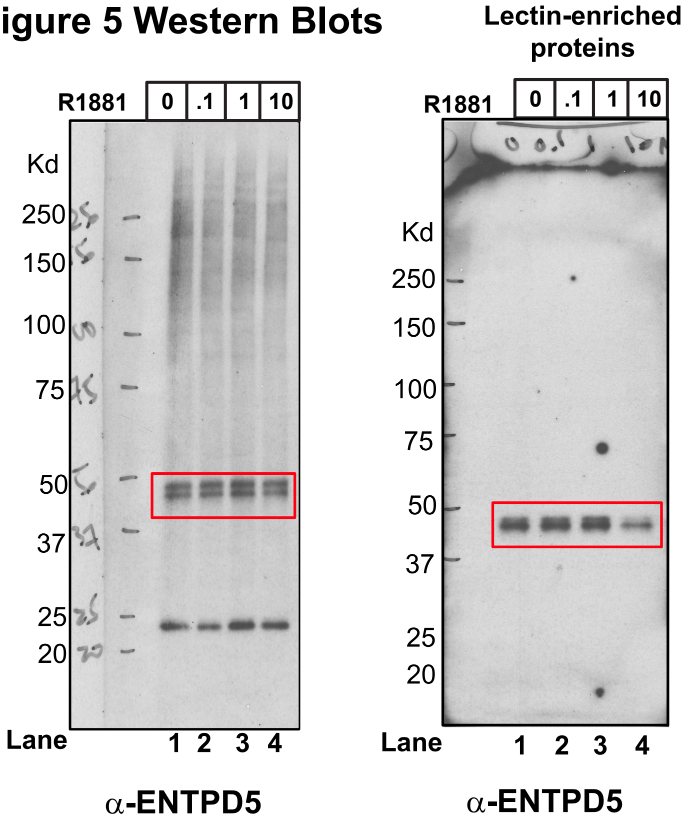

Supplemental Fig. 2

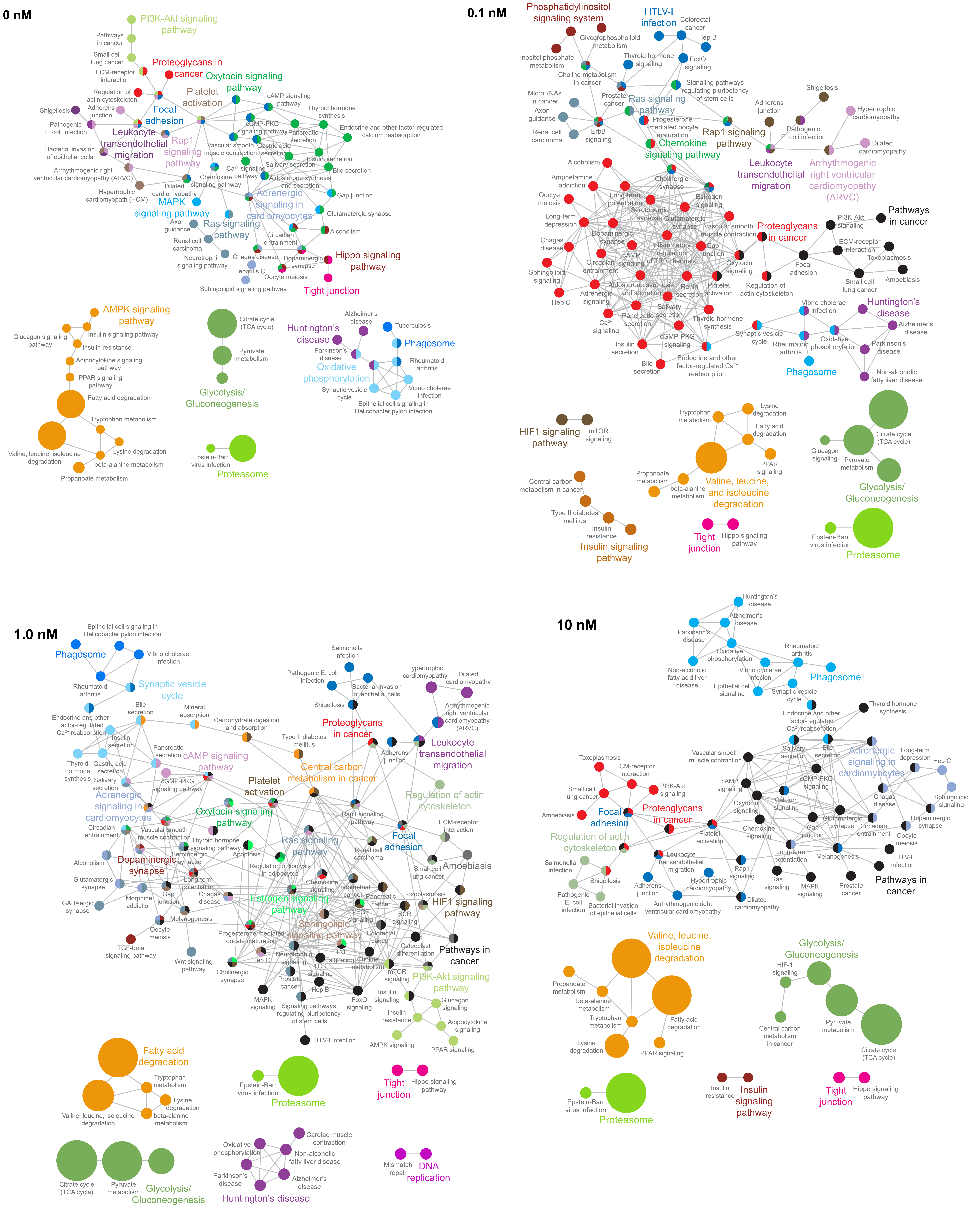

Supplemental Fig. 3

Supplemental Figure 3. Network visualization of enriched pathways in androgen-responsive glycoproteins in LNCaP cells. Protein interaction networks were built in Cytoscape, with ontology and annotation information downloaded through the Cytoscape interface. Detailed descriptions of the steps used in the processing and filtering of data are detailed in the supplemental methods section.

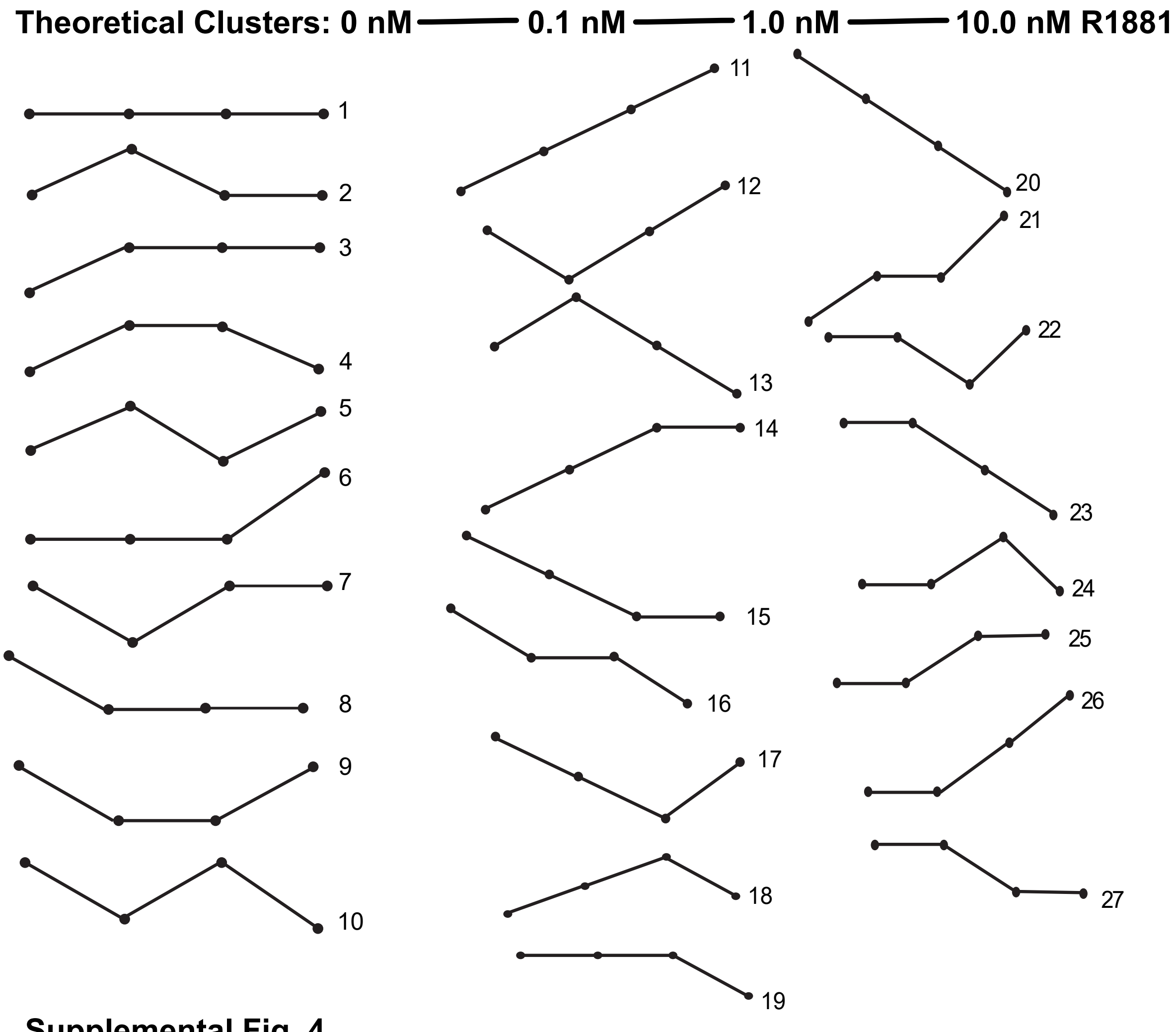

Supplemental Fig. 4

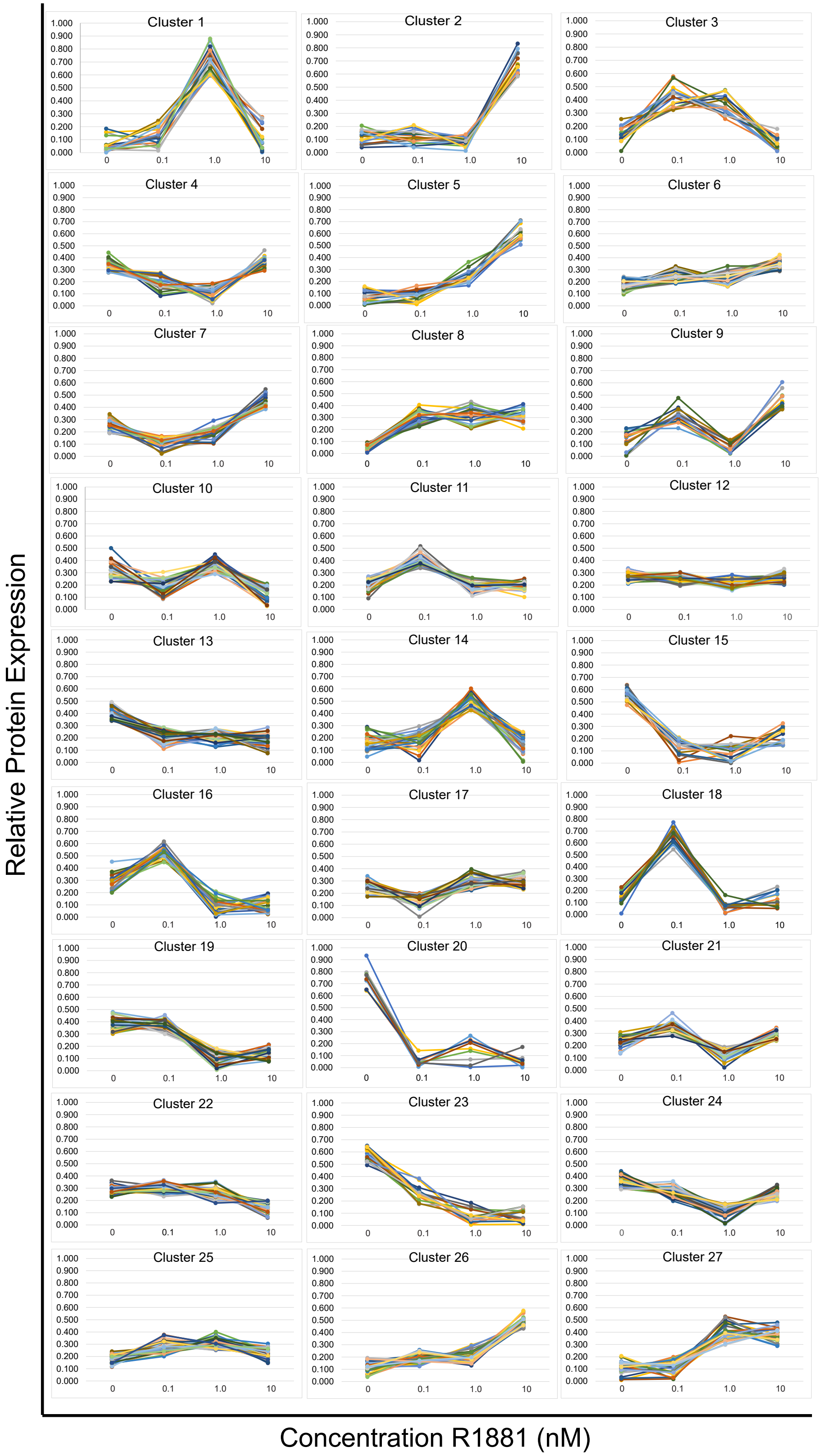

Supplemental Fig. 5

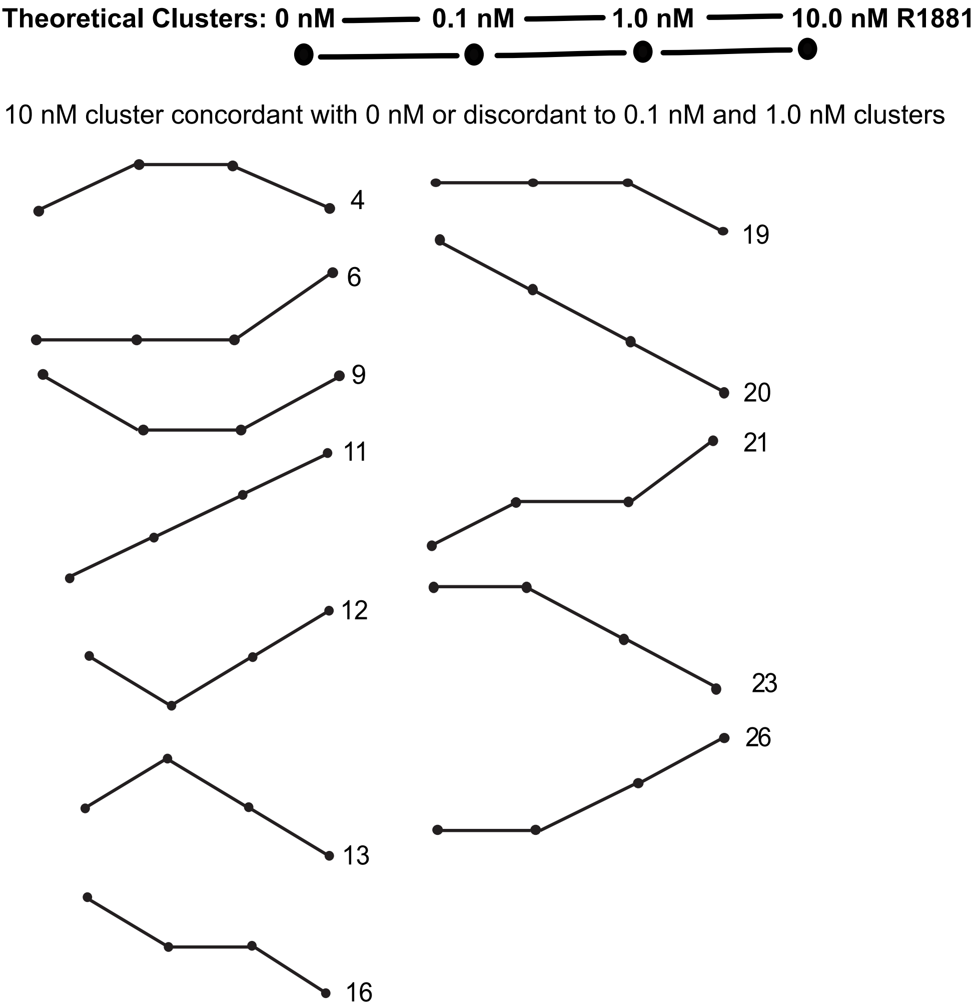

Supplemental Fig. 6

Normal Adjacent tissues

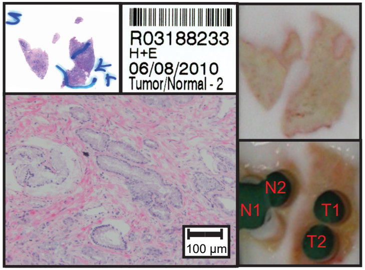

Pt. Age: NK
Gleason: 3+4=7
PSA: 5.9
Stage: II
% Tumor: NK
Full Weight: NK
N Cut Weight: 1.37 g
T Cut Weight: 1.36 g

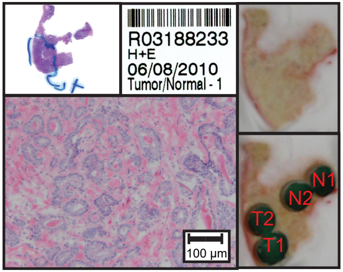

Pt. Age: NK
Gleason: 3+4=7
PSA: 5.9
Stage: II
% Tumor: NK
Full Weight: NK
N Cut Weight: N: 1.55 g
T Cut Weight: 1.55g

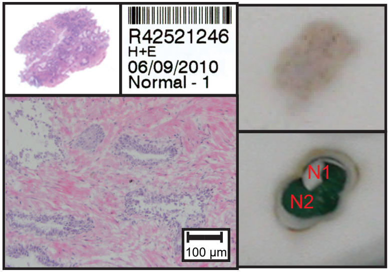

Pt. Age:70
Gleason: 4+5=9
PSA: 37.43
Stage: III
% Tumor: 15
Full Weight: 59 g
N Cut Weight: 1.59 g
T Cut Weight: 3.08 g

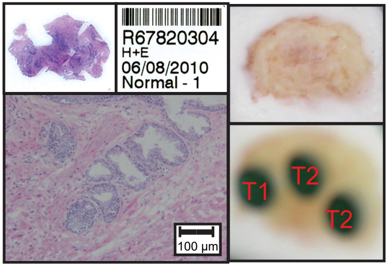

Pt. Age: 51
Gleason: 4+3=7
PSA: 7.18
Stage: II
% Tumor: NK
Full Weight: 44.65
N Cut Weight: 1.55 g
T Cut Weight: 1.57 g

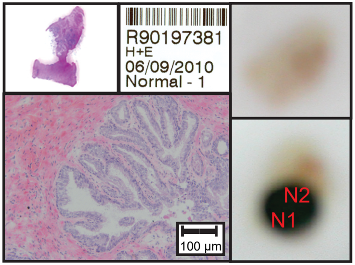

Pt. Age: 61
Gleason: 5+5=10
PSA: 7.77
Stage: III
% Tumor: NK
FullWeight: 45.6
N Cut Weight: 1.53 g
T Cut Weight: 1.53 g

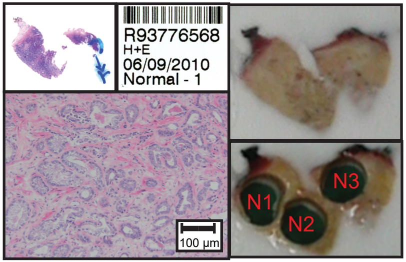

Pt. Age: NK
Gleason: 3+4=7
PSA: 4.08
Stage: II
% Tumor: NK
Full Weight: 39.55 g
N Cut Weight: 2.28 g
T Cut Weight: 2.29 g

PANEL 1

Supplemental Fig. 7

Localized disease

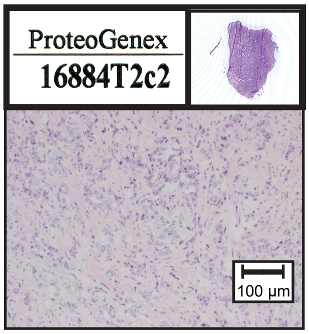

Pt. Age: 61
Tissue Type: Tumor
Gleason: 3+4=7
PSA: 13.5
Stage: II
% Tumor: 100
Sample Weight: 0.40 g

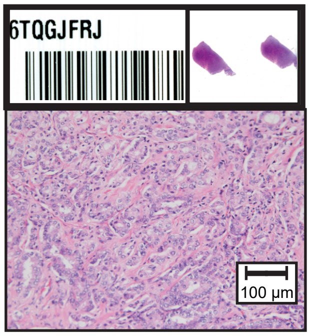

Pt. Age: 71
Tissue Type: Tumor
Gleason: 4+3=7
PSA: 13
Stage: II
% Tumor: 80
Sample Weight: 0.116 g

GT55Q
Not Provided

Pt. Age: 66
Tissue Type: Tumor
Gleason: 6
PSA: 5
Stage: II
% Tumor: 80
Sample Weight: 0.075 g

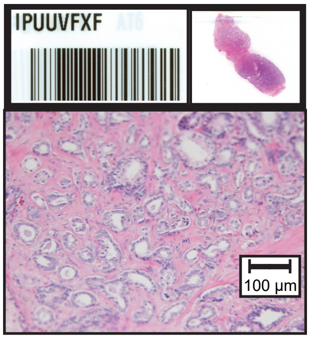

Pt. Age: 63
Tissue Type: Tumor
Gleason: 7
PSA: 3
Stage: II
% Tumor: 55
Sample Weight: 0.468 g

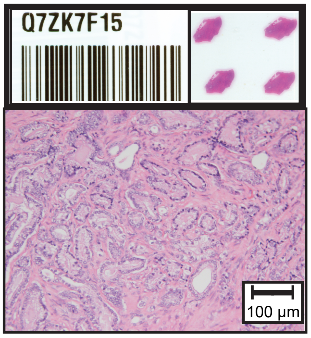

Pt. Age: 69
Tissue Type: Tumor
Gleason: 7
PSA: 19
Stage: II
% Tumor: 80
Sample Weight: 0.076 g

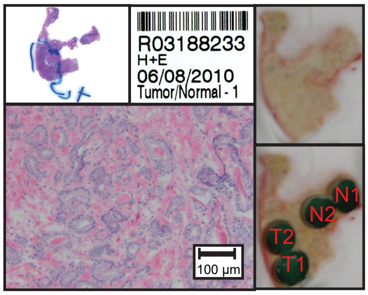

Pt. Age: NK
Gleason: 3+4=7
PSA: 5.9
Stage: II
% Tumor: NK
Full Weight: NK
N Cut Weight: 1.55 g
T Cut Weight: 1.55g

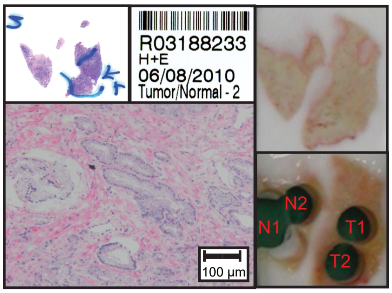

Pt. Age: NK
Gleason: 3+4=7
PSA: 5.9
Stage: II
% Tumor: NK
Full Weight: NK
N Cut Weight: 1.37 g
T Cut Weight: 1.36 g

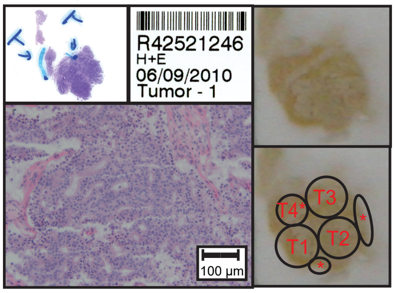

Pt. Age:70
Gleason: 4+5=9
PSA: 37.43
Stage: III
% Tumor: 15
Full Weight: 59 g
N Cut Weight: 1.59 g
T Cut Weight: 3.08 g

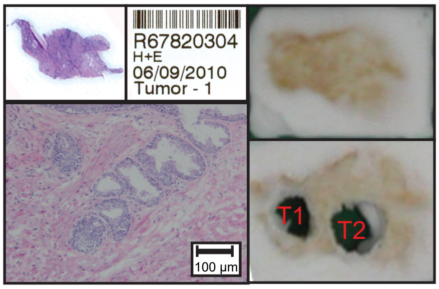

Pt. Age: 51
Gleason: 4+3=7
PSA: 7.18
Stage: II
% Tumor: NK
Full Weight: 44.65
N Cut Weight: 1.55 g
T Cut Weight: 1.57 g

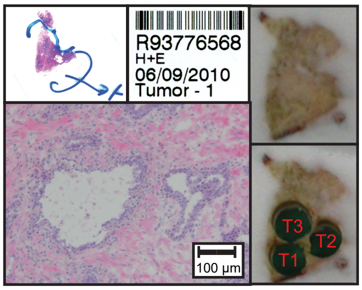

Pt. Age: NK
Gleason: 3+4=7
PSA: 4.08
Stage: II
% Tumor: NK
Full Weight: 39.55 g
N Cut Weight: 2.28 g
T Cut Weight: 2.29 g

PANEL 2

BPH/Normal Adjacent tissues

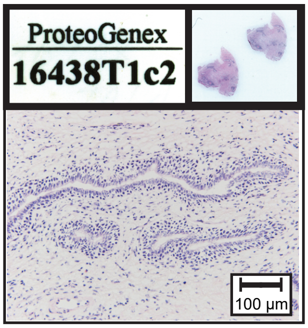

Pt Age: 66
Tissue Type: BPH
Sample Weight: 0.56 g

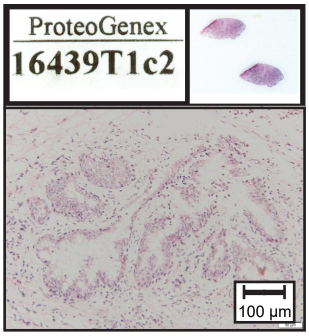

Pt Age: 60
Tissue Type: BPH
Sample Weight: 0.511 g

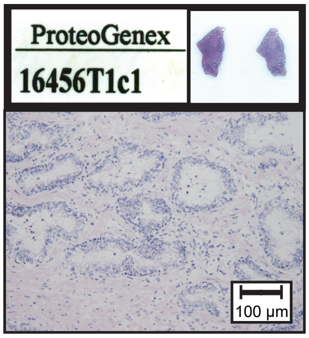

Pt Age:61
Tissue Type: BPH
Sample Weight: 0.809 g

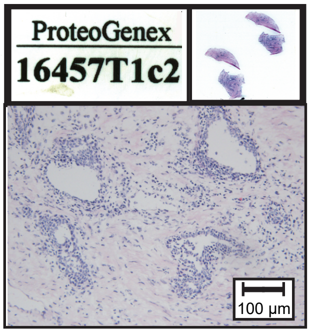

Pt Age: 67
Tissue Type: BPH
Sample Weight: 0.819 g

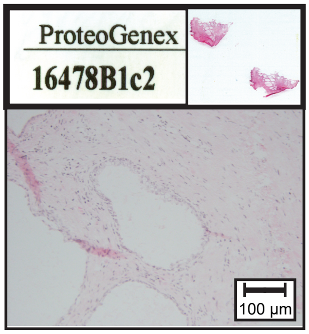

Pt Age: 61
Tissue Type: BPH
Sample Weight: 0.723 g

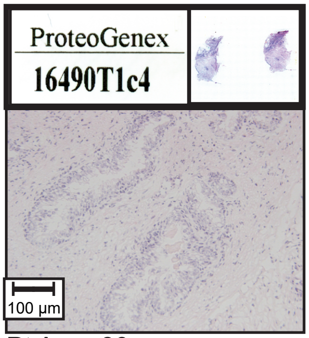

Pt Age: 60
Tissue Type: BPH
Sample Weight: 0.656 g

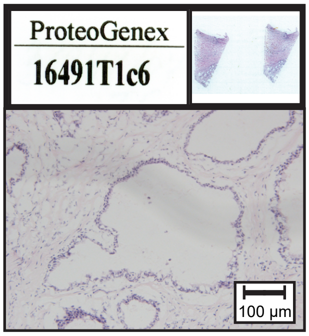

Pt Age: 60
Tissue Type: BPH
Sample Weight: 0.793 g

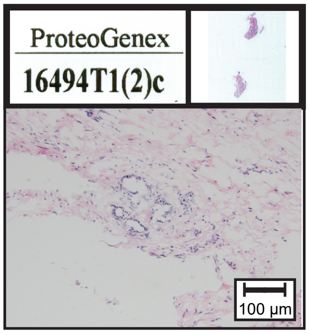

Pt Age: 65
Tissue Type: BPH
Sample Weight: 0.606 g

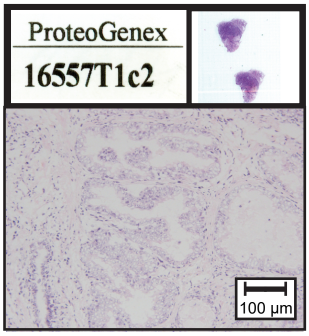

Pt Age: 60
Tissue Type: BPH
Sample Weight:0.602 g

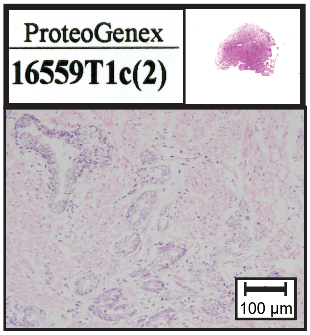

Pt Age: 66
Tissue Type: BPH
Sample Weight: 0.851

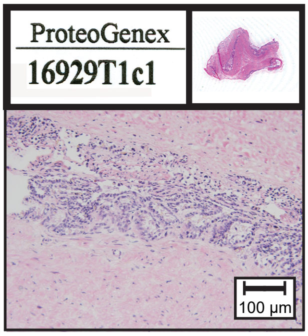

Pt. Age: 54
Tissue Type: NAT
Sample Weight: 0.79 g

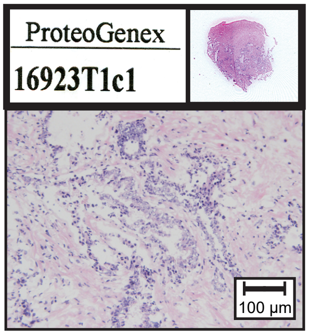

Pt. Age: 65
Tissue Type: NAT
Sample Weight:0.44 g

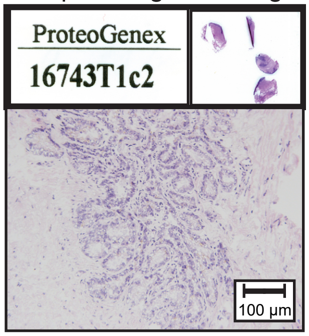

Pt. Age: 62
Tissue Type: NAT
Sample Weight: 0.83 g

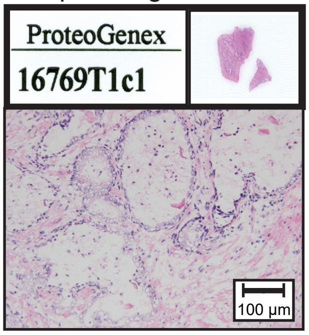

Pt. Age: 60
Tissue Type: NAT
Sample Weight: 0.68 g

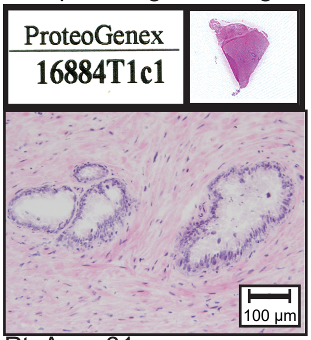

Pt. Age: 61
Tissue Type: NAT
Sample Weight: 0.44 g

PANEL 3

Localized and metastatic disease

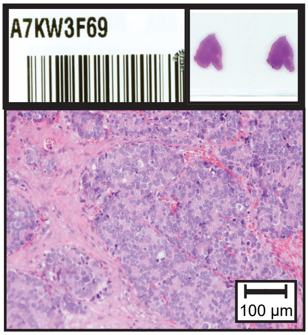

Pt. Age: 82
Tissue Type: Tumor
Gleason: NK
PSA: NK
Stage: NK
% Tumor: 95
Sample Weight: 0.804 g

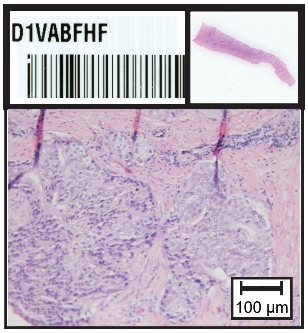

Pt. Age 62:
Tissue Type: Tumor
Gleason: 9
PSA: 2
Stage: IV
% Tumor: 80
Sample Weight: 0.359 g

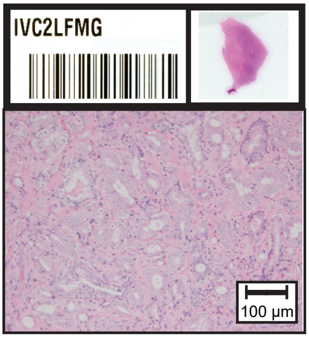

Pt. Age: 72
Tissue Type: Tumor
Gleason: 6
PSA: NK
Stage: III
% Tumor: 65
Sample Weight: 0.129 g

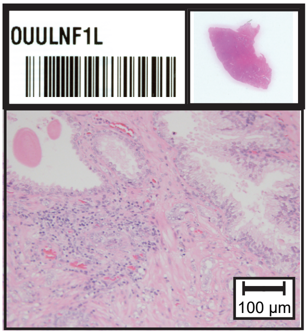

Pt. Age: 60
Tissue Type: Tumor
Gleason: 7
PSA: 34
Stage: IV
% Tumor: 50
Sample Weight: 0.25 g

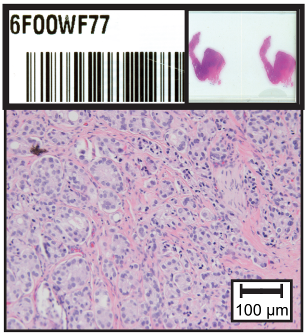

Pt. Age: 61
Tissue Type: Tumor
Gleason: 5+4=9
PSA: 11
Stage: III
% Tumor: 55
Sample Weight: 0.308 g

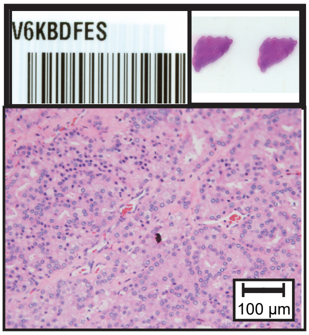

Pt. Age: 71
Tissue Type: Tumor
Gleason: 9
PSA: NK
Stage: III
% Tumor: 90
Sample Weight: 0.198 g

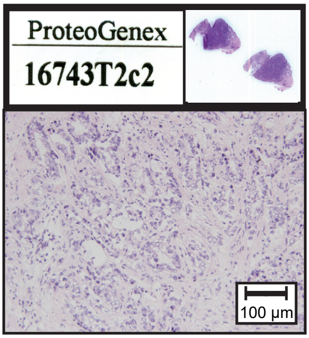

Pt. Age: 62
Tissue Type: Tumor
Gleason: 3+4=7
PSA: 4.89
Stage: III
% Tumor: 60
Sample Weight: 0.82 g

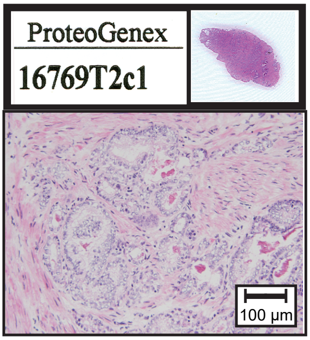

Pt. Age: 60
Tissue Type: Tumor
Gleason: 3+3=6
PSA: NK
Stage: III
% Tumor: 70
Sample Weight: 0.87 g

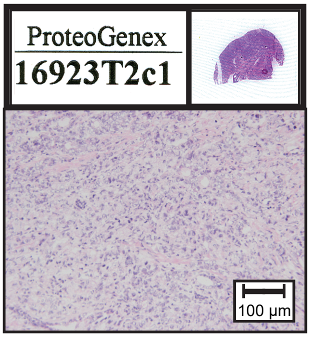

Pt. Age: 65
Tissue Type: Tumor
Gleason: 4+3=7
PSA: NK
Stage: III
% Tumor: 100
Sample Weight: 0.43 g

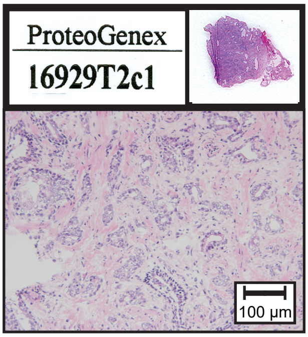

Pt. Age: 54
Tissue Type: Tumor
Gleason: 3+4=7
PSA:5.2
Stage: III
% Tumor: 75
Sample Weight: 0.52 g

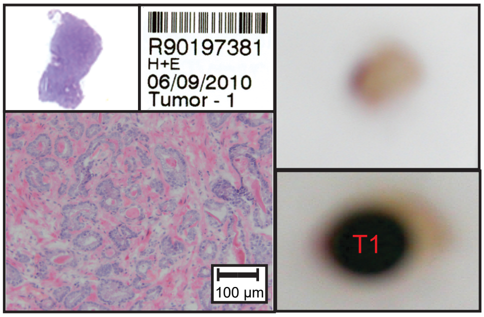

Pt. Age: 61
Gleason: 5+5=10
PSA: 7.77
Stage: III
% Tumor: NK
FullWeight: 45.6
N Cut Weight: 1.53 g
T Cut Weight: 1.53 g

PANEL 4

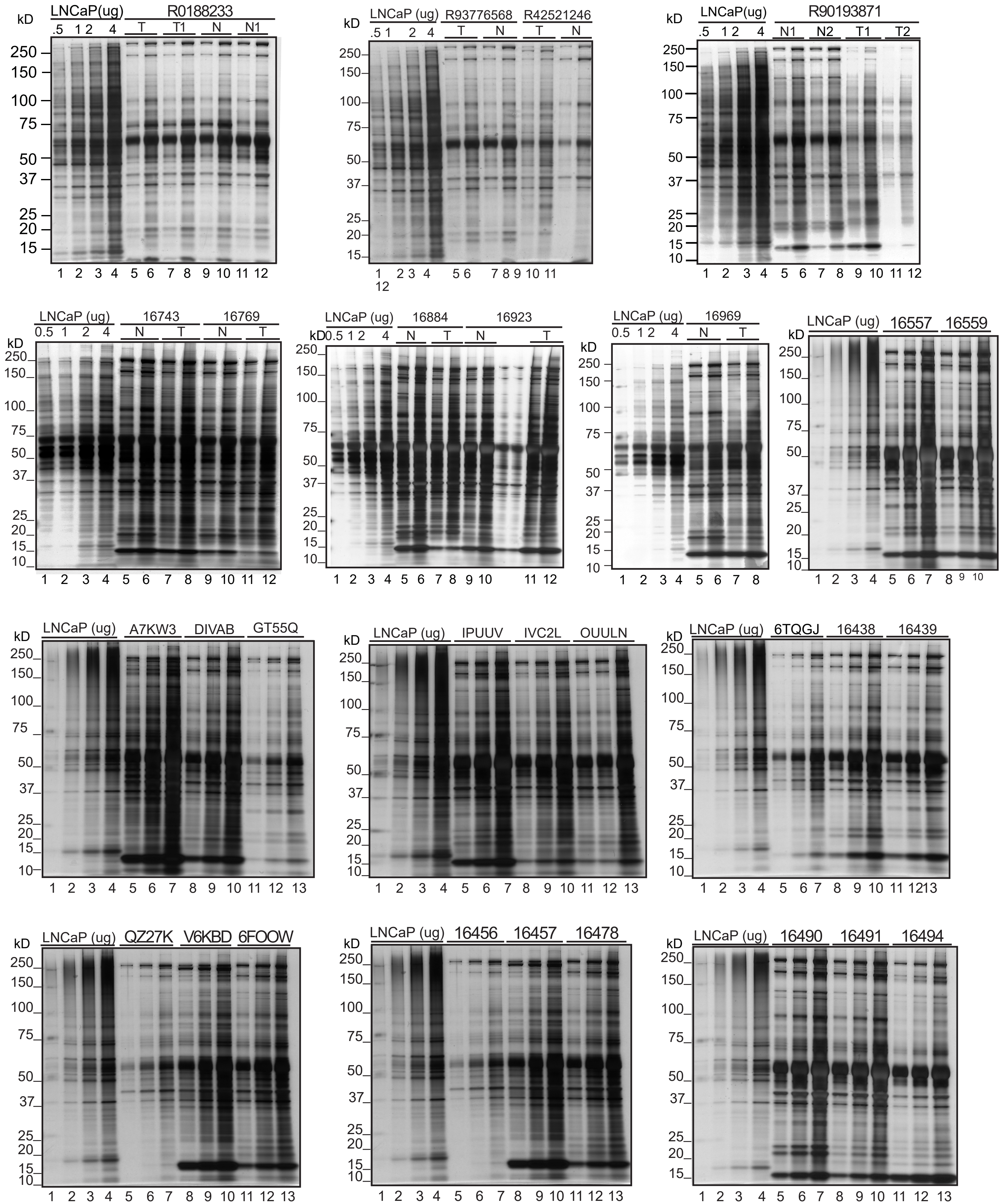

Supplemental Fig. 8

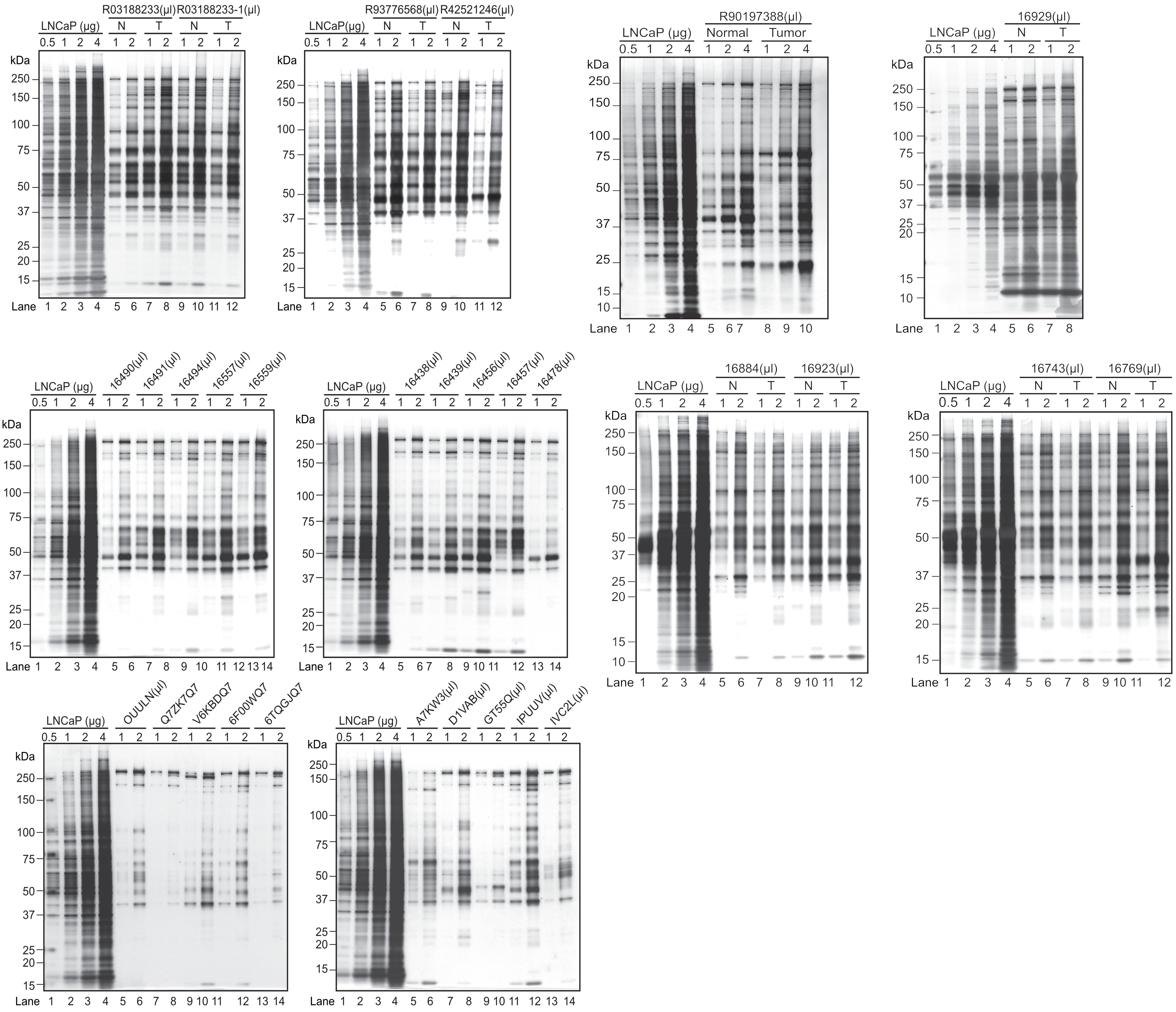

Supplemental Fig. 9

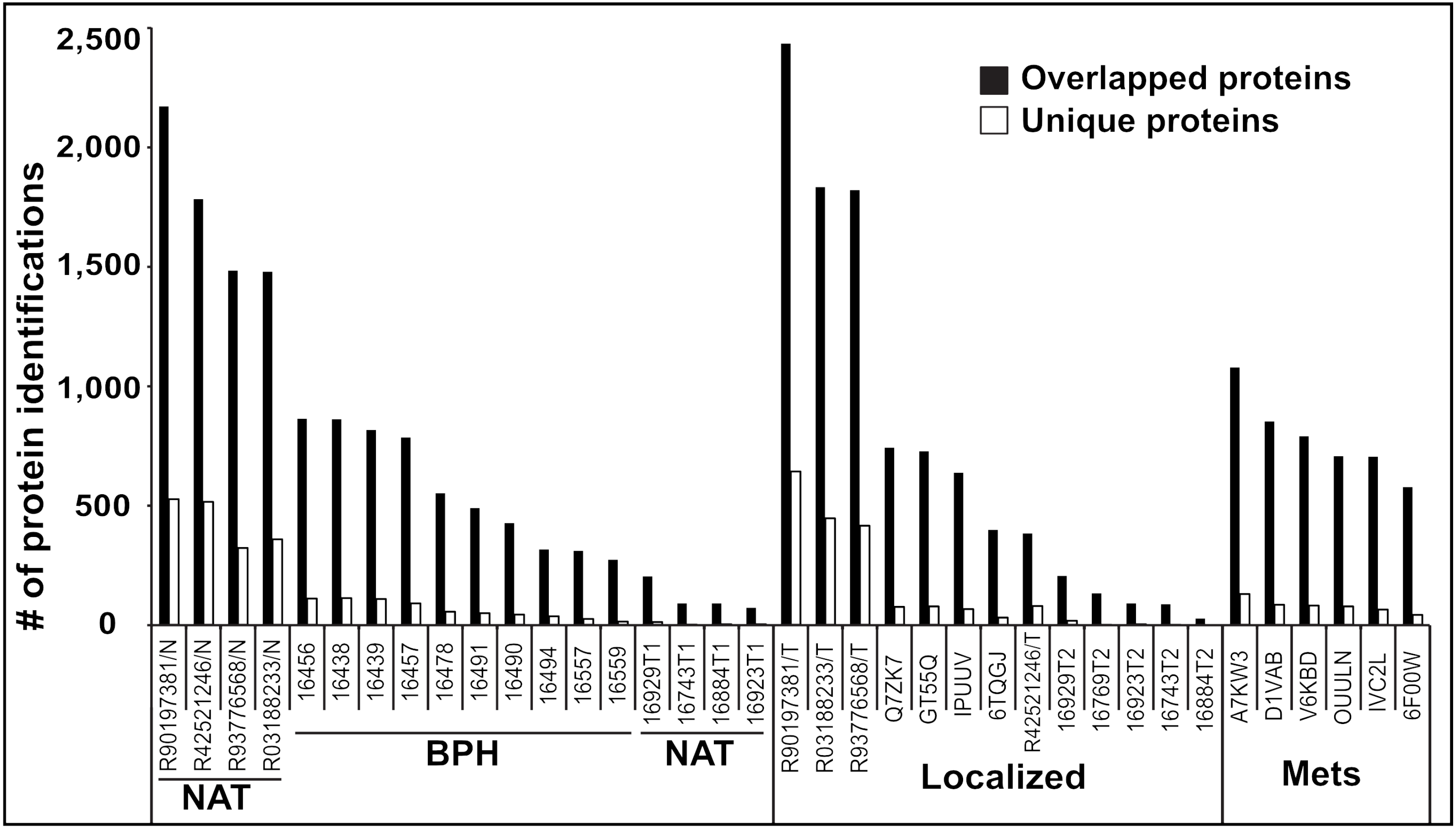

Supplemental Fig. 10

Biological replicate #2

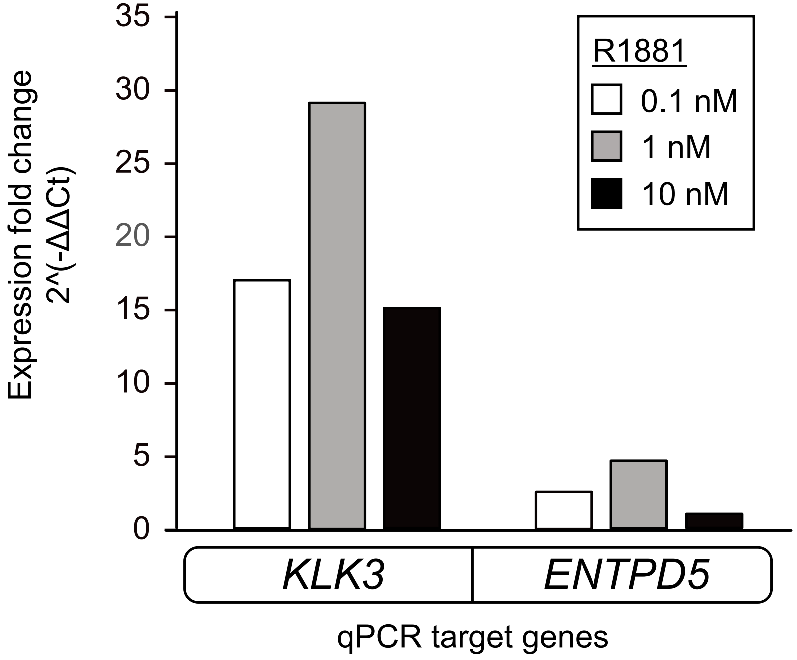

Biological replicate #3

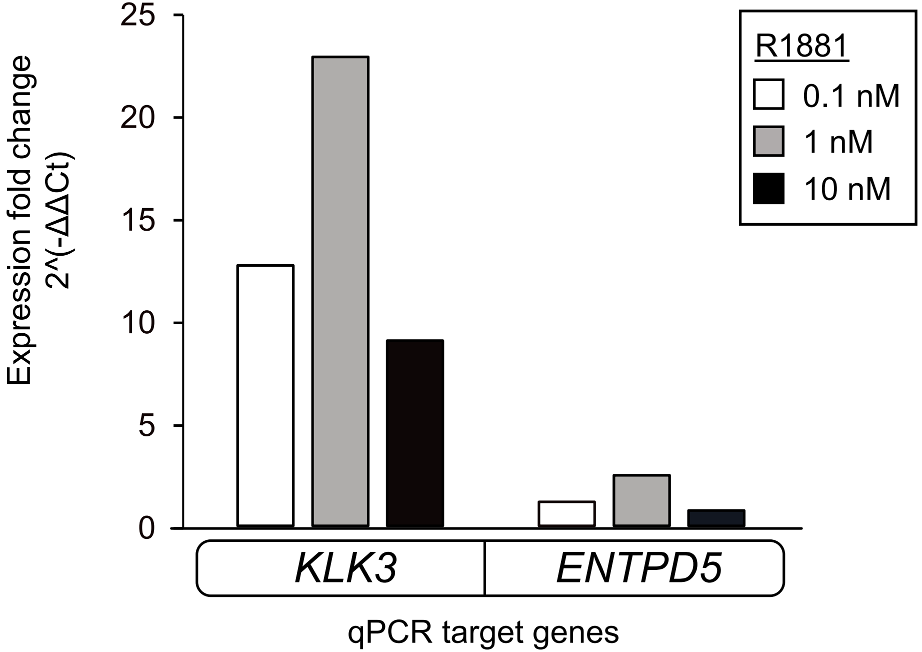

Supplemental Fig. 11

A

chr14:74,482,139-74,482,531/ ENTPD5 Intron #2

CTCAGGTGAT***CCTCTGAACTCAGCC***TCCTGAGT***AGCTGGAACTACAGG***TACATGG***CCACC*** 60
ACACCCAGCTAATTTTTGTATTTTTTGTAAAGACAGGGTTTTG***CCATGTTGTCCAGAC***TG 120
GTCTTGAACTCCTGGGCTTAAGCAATCCGTCTGCCTTGGCCTCCCAAAGTGTTAGGATTA 180
CAGGCATGAA***CCACCATGACCAGCC***AGAAATTCTATCTATTTTGATACATAATAGTTCTA 240
AATTTTATATATGGCTTGTTGGACAAAATTAACATCACTTGGCAAAAAACAGAGAATTAA 300
ATTCAGGAAAAAAAGAAAACCACCATTCAGGAACATTTGAAATACATTTATAAATTTAAG 360
CTAGTGGAGATATTA 375

chr14:74,458,755-74,459,147/ ENTPD5 Intron #6

CCCATTTC***CCAGGCCCTTCAGCT***CTAACAAAATACTTCGTATATTTGGAGTACAGATTAA 60
GGATTCTCAATCATCTCAACTTTGTTGAGTATCTAGGGTTTATCAAAATGTCTCCCTCCC 120
CACAG 125

Name	Strand	Start	*p*-value	Sites
ENTPD5 Intron #2	+	56	9.37e-8	AGGTACATGG CCACCACACCCAGCT AATTTTTGTA
ENTPD5 Intron #2	+	191	1.76e-7	CAGGCATGAA CCACCATGACCAGCC AGAAATTCTA
ENTPD5 Intron #2	-	34	5.42e-7	TGGCCATGTA CCTGTAGTTCCAGCT ACTCAGGAGG
ENTPD5 Intron #6	+	9	1.39e-6	CCCATTTC CCAGGCCCTTCAGCT CTAACAAAAT
ENTPD5 Intron #2	-	121	1.79e-6	TTGCTTAAGC CCAGGAGTTCAAGAC CAGTCTGGAC
ENTPD5 Intron #2	+	104	3.54e-6	CAGGGTTTTG CCATGTTGTCCAGAC TGGTCTTGAA
ENTPD5 Intron #2	+	11	8.53e-6	CTCAGGTGAT CCTCTGAACTCAGCC TCCTGAGTAG

Forward **Reverse complement**

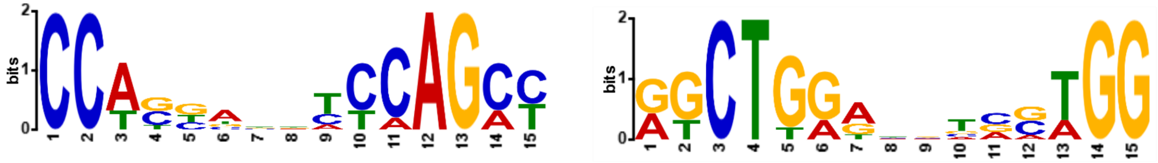

Motif: CCASBANNYCCAGCY

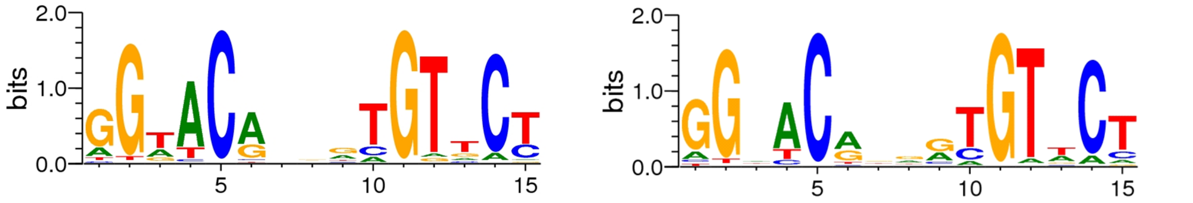

AR:M00481: GGWACRNNNTGTNCY **AR:M01201:** GGNACRNNRTGTDCT

B

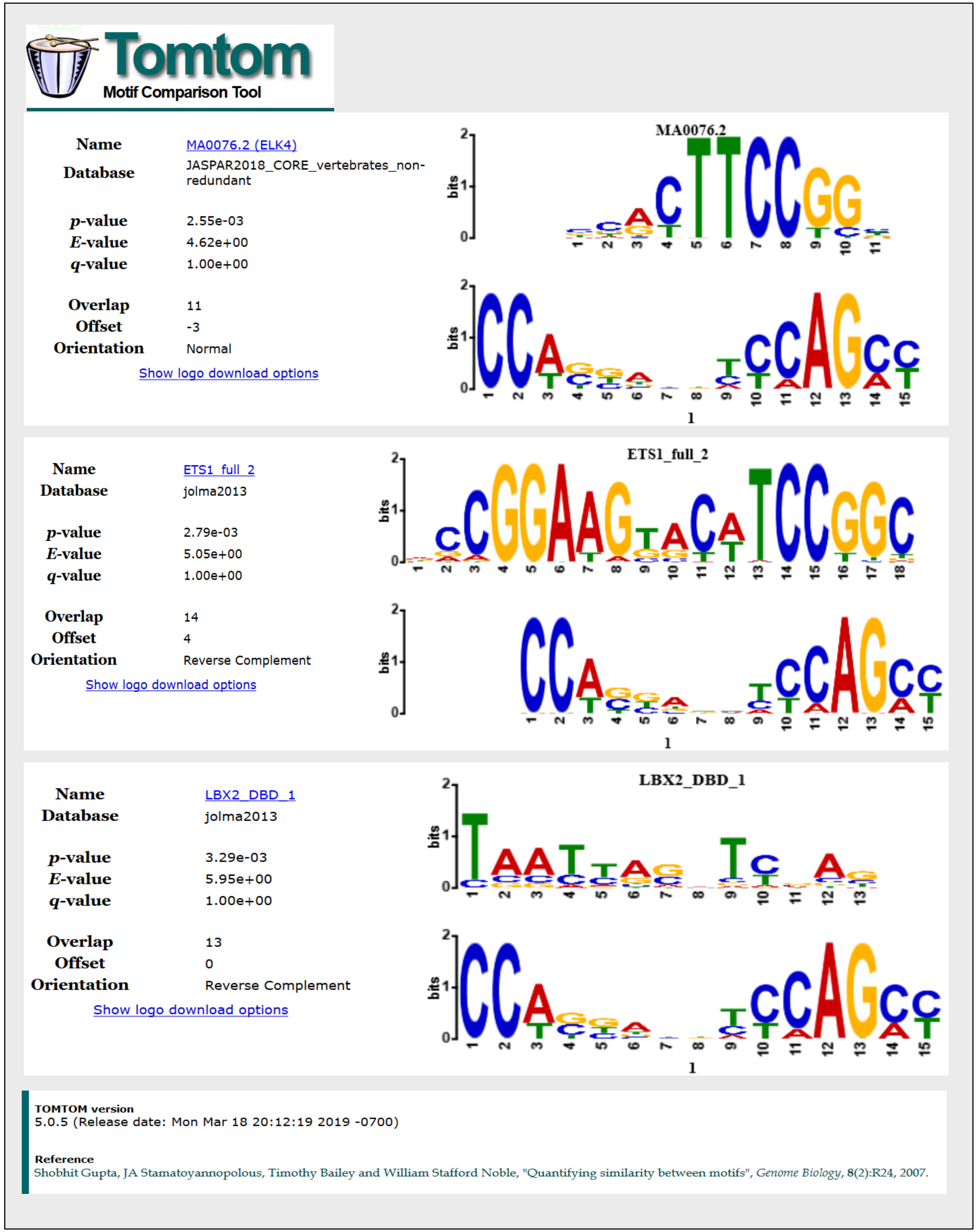

Supplemental Fig. 12

Supplemental Fig. 13

Protocol for Tissue Proteomics

-Export the data from Spectrum Mill using “PPonSM” setting

-Open the file using Excel

-The data will look scrambled. To un-scramble, select the first column, “A”. Then go over to the “Data” tab on Excel , -> “Text to Columns” -> Select “Delimited” -> Next -> Check “Semicolon”-> Finish.

-Reorganize the columns so the total intensity of all the samples, accession number, and entry name are together

-End result will look something like this

Clipboard | Font | Alignment | Number | Styl

AL1 | f_x | entry_name

	AB	AC	AD	AE	AF	AG	AH	AI	AJ	AK	AL	AM
1	TissuePr oteomic s/Q7ZK7 totalInt ensity	TissuePr oteomic s/R0318 8233/N totalInt ensity	TissuePr oteomic s/R0318 8233/T totalInt ensity	TissuePr oteomic s/R4252 1246/N totalInt ensity	TissuePr oteomic s/R4252 1246/T totalInt ensity	TissuePr oteomic s/R9019 7381/N totalInt ensity	TissuePr oteomic s/R9019 7381/T totalInt ensity	TissuePr oteomic s/R9377 6568/N totalInt ensity	TissuePr oteomic s/R9377 6568/T totalInt ensity	TissuePr oteomic s/V6KBD totalInt ensity	entry_n ame	accessi on_num ber
2	3.90E+08	1.62E+08	1.87E+08	5.68E+07	5.62E+05	7.22E+08	4.36E+08	2.01E+08	1.32E+08	8.24E+07	Filamin-/	P21333
3	9.10E+07	2.15E+07	4.54E+06	1.19E+06	5.18E+07	1.08E+07	3.23E+08	1.31E+06	1.48E+07	4.39E+08	Fatty acic	P49327
4	7.06E+05	1.06E+08	1.31E+08	2.93E+07	2.17E+06	1.80E+08	1.58E+08	2.57E+08	1.53E+08	2.48E+07	Complem	P01024
5	3.88E+07	1.14E+06	1.16E+06	1.88E+05	0.00E+00	2.49E+07	7.33E+06	3.55E+06	2.35E+06	3.86E+06	Talin-1	Q9Y490
6	1.12E+07	1.61E+09	9.41E+08	1.97E+08	2.84E+07	9.48E+08	4.77E+08	3.12E+09	1.13E+09	4.95E+07	Serotran:	P02787
7	7.73E+07	4.85E+08	1.94E+08	1.06E+08	6.04E+07	1.12E+09	5.47E+08	2.47E+09	6.30E+08	1.31E+08	Serum al	P02768
8	3.69E+06	7.32E+07	8.97E+07	1.38E+08	1.09E+05	1.28E+08	9.49E+06	2.87E+08	9.90E+07	3.72E+07	Alpha-2-ı	P01023
9	4.56E+07	5.08E+07	6.50E+07	4.55E+07	6.21E+05	2.78E+08	2.63E+08	5.04E+07	6.87E+07	4.95E+06	Isoform :	P12814-3
10	1.32E+06	1.16E+07	3.33E+08	5.44E+07	0.00E+00	2.75E+08	7.72E+07	1.09E+08	2.61E+08	0.00E+00	Lactotrar	P02788
11	1.86E+06	2.89E+05	0.00E+00	2.40E+05	0.00E+00	7.93E+04	2.22E+07	1.05E+05	1.84E+06	5.23E+06	Isoform :	Q14980-2
12	2.83E+06	1.19E+06	3.36E+06	2.17E+06	3.88E+04	1.56E+07	5.24E+07	1.78E+07	2.46E+07	1.41E+05	Spectrin	A6NG51
13	9.23E+05	0.00E+00	2.45E+05	9.06E+04	5.39E+04	2.27E+06	2.62E+07	0.00E+00	6.23E+05	2.96E+06	Cytoplasi	Q14204
14	3.25E+05	2.29E+06	1.94E+06	1.12E+06	0.00E+00	2.61E+07	1.87E+07	1.16E+06	5.87E+06	6.19E+05	Neurobla	Q09666
15	0.00E+00	0.00E+00	0.00E+00	4.50E+05	8.04E+04	0.00E+00	2.71E+06	1.01E+06	1.44E+05	1.51E+06	Collagen	D6RGG3

Tip: Renaming the columns will be easier on the eyes and also later proteomics

1. Grabbing Gene Names

-Go the uniprot.org

-Select the “Retrieve” Tab on the top

-Select the column the contains accession numbers on your Excel file and copy them into the “Uniprot Identifiers” Box. Then click “Retrieve”

-Once completed, download and open the file in Excel

-Select the first column. On the “Home” tab in the Excel, go “conditional formatting” -> “highlight cell rules” -> “text that contains”. Type “>” in the box and then hit OK.

-Sort the column by color

Click line 1 -> “Sort & Filter”-> “Filter”

Click on the arrow box the appears. Select “Sort by colors” -> Select color of highlighted cells

End result should look like this:

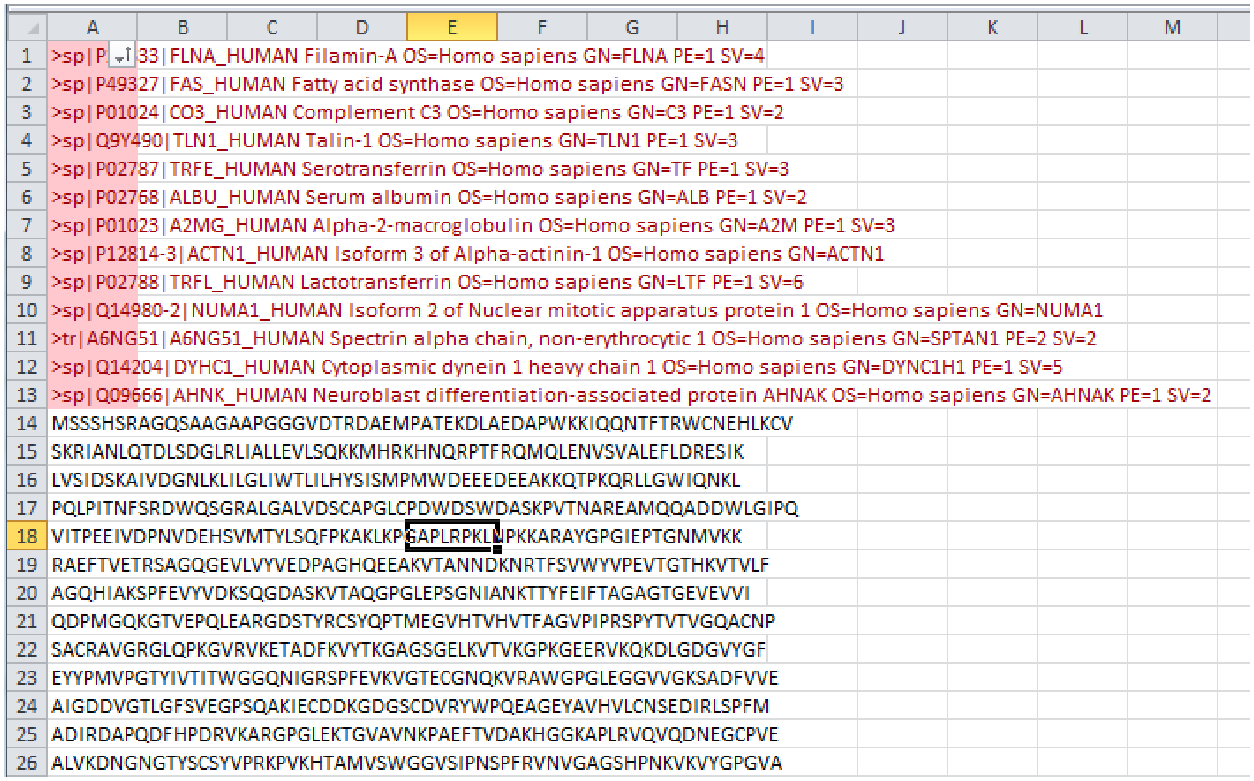

	A
1	>sp|P[illegible]33|FLNA_HUMAN Filamin-A OS=Homo sapiens GN=FLNA PE=1 SV=4
2	>sp|P49327|FAS_HUMAN Fatty acid synthase OS=Homo sapiens GN=FASN PE=1 SV=3
3	>sp|P01024|CO3_HUMAN Complement C3 OS=Homo sapiens GN=C3 PE=1 SV=2
4	>sp|Q9Y490|TLN1_HUMAN Talin-1 OS=Homo sapiens GN=TLN1 PE=1 SV=3
5	>sp|P02787|TRFE_HUMAN Serotransferrin OS=Homo sapiens GN=TF PE=1 SV=3
6	>sp|P02768|ALBU_HUMAN Serum albumin OS=Homo sapiens GN=ALB PE=1 SV=2
7	>sp|P01023|A2MG_HUMAN Alpha-2-macroglobulin OS=Homo sapiens GN=A2M PE=1 SV=3
8	>sp|P12814-3|ACTN1_HUMAN Isoform 3 of Alpha-actinin-1 OS=Homo sapiens GN=ACTN1
9	>sp|P02788|TRFL_HUMAN Lactotransferrin OS=Homo sapiens GN=LTF PE=1 SV=6
10	>sp|Q14980-2|NUMA1_HUMAN Isoform 2 of Nuclear mitotic apparatus protein 1 OS=Homo sapiens GN=NUMA1
11	>tr|A6NG51|A6NG51_HUMAN Spectrin alpha chain, non-erythrocytic 1 OS=Homo sapiens GN=SPTAN1 PE=2 SV=2
12	>sp|Q14204|DYHC1_HUMAN Cytoplasmic dynein 1 heavy chain 1 OS=Homo sapiens GN=DYNC1H1 PE=1 SV=5
13	>sp|Q09666|AHNK_HUMAN Neuroblast differentiation-associated protein AHNAK OS=Homo sapiens GN=AHNAK PE=1 SV=2
14	MSSSHSRAGQSAAGAAPGGGVDTRDAEMPATEKDLAEDAPWKKIQQNTFTRWCNEHLKCV
15	SKRIANLQTDLSDGLRLIALLEVLSQKKMHRKHNQRPTFRQMQLENVSVALEFLDRESIK
16	LVSIDSKAIVDGNLKLILGLIWTLILHYSISMPMWDEEEDEEAKKQTPKQRLLGWIQNKL
17	PQLPITNFSRDWQSGRALGALVDSCAPGLCPDWDSWDASKPVTNAREAMQQADDWLGIPQ
18	VITPEEIVDPNVDEHSVMTYLSQFPKAKLKPGAPLRPKLNPKKARAYGPGIEPTGNMVKK
19	RAEFTVETRSAGQGEVLVYVEDPAGHQEEAKVTANNDKNRTFSVWYVPEVTGTHKVTVLF
20	AGQHIAKSPFEVYVDKSQGDASKVTAQGPGLEPSGNIANKTTYFEIFTAGAGTGEVEVVI
21	QDPMGQKGTVEPQLEARGDSTYRCSYQPTMEGVHTVHVTFAGVPIPRSPYTVTVGQACNP
22	SACRAVGRGLQPKGVRVKETADFKVYTKGAGSGELKVTVKGPKGEERVKQKDLGDGVYGF
23	EYYPMVPGTYIVTITWGGQNIGRSPFEVKVGTECGNQKVRAWGPGLEGGVVGKSADFVVE
24	AIGDDVGTLGFSVEGPSQAKIECDDKGDGSCDVRYWPQEAGEYAVHVLCNSEDIRLSPFM
25	ADIRDAPQDFHPDRVKARGPGLEKTGVAVNKPAEFTVDAKHGGKAPLRVQVQDNEGCPVE
26	ALVKDNGNGTYSCSYVPRKPVKHTAMVSWGGVSIPNSPFRVNVGAGSHPNKVKVYGPGVA

-Select all the highlighted cell and place them in a new sheet

-Now you want to separate cells so the accession number and Gene Name (“GN”) are separated.

(Delimit by “|” -> “=”)

-Delete all the columns except for the accession number and gene name

End with this:

1	P21333	FLNA
2	P49327	FASN
3	P01024	C3
4	Q9Y490	TLN1
5	P02787	TF
6	P02768	ALB
7	P01023	A2M
8	P12814-3	ACTN1
9	P02788	LTF
10	Q14980-2	NUMA1
11	A6NG51	SPTAN1
12	Q14204	DYNC1H1
13	Q09666	AHNAK
14		
15		

-Copy both columns into your spectrum mill results

End should look like this

	AG	AH	AI	AJ	AK	AL	AM	AN	AO	AP	AQ
	TissueProteomics/R90197381/N totalIntensity	TissueProteomics/R90197381/T totalIntensity	TissueProteomics/R93776568/N totalIntensity	TissueProteomics/R93776568/T totalIntensity	TissueProteomics/V6KBD totalIntensity	entry_name	accession_number				
5	7.22E+08	4.36E+08	2.01E+08	1.32E+08	8.24E+07	Filamin-A	P21333		P21333	FLNA	
7	1.08E+07	3.23E+08	1.31E+06	1.48E+07	4.39E+08	Fatty acid	P49327		P49327	FASN	
6	1.80E+08	1.58E+08	2.57E+08	1.53E+08	2.48E+07	Complem	P01024		P01024	C3	
0	2.49E+07	7.33E+06	3.55E+06	2.35E+06	3.86E+06	Talin-1	Q9Y490		Q9Y490	TLN1	
7	9.48E+08	4.77E+08	3.12E+09	1.13E+09	4.95E+07	Serotransi	P02787		P02787	TF	
7	1.12E+09	5.47E+08	2.47E+09	6.30E+08	1.31E+08	Serum alb	P02768		P02768	ALB	
5	1.28E+08	9.49E+06	2.87E+08	9.90E+07	3.72E+07	Alpha-2-m	P01023		P01023	A2M	
5	2.78E+08	2.63E+08	5.04E+07	6.87E+07	4.95E+06	Isoform 3	P12814-3		P12814-3	ACTN1	
0	2.75E+08	7.72E+07	1.09E+08	2.61E+08	0.00E+00	Lactotrans	P02788		P02788	LTF	
0	7.93E+04	2.22E+07	1.05E+05	1.84E+06	5.23E+06	Isoform 2	Q14980-2		Q14980-2	NUMA1	
4	1.56E+07	5.24E+07	1.78E+07	2.46E+07	1.41E+05	Spectrin a	A6NG51		A6NG51	SPTAN1	
4	2.27E+06	2.62E+07	0.00E+00	6.23E+05	2.96E+06	Cytoplasm	Q14204		Q14204	DYNC1H1	
0	2.61E+07	1.87E+07	1.16E+06	5.87E+06	6.19E+05	Neurobla:	Q09666		Q09666	AHNAK	

In the ideal world, you can see the accession numbers align up exactly across, that is usually not the case if you are dealing with huge lists. To make the aligned:

-Select both accession number columns -> “Highlight cell rules”-> “Duplicate Values”

-Sort the first accession number column by color

Now they should be aligned

Notes:

-You will get blanks, numbers and dates

Blanks means Uniprot couldn't identify the Accession Number. The Accession number has changed or been deleted

Fix: look them up individually

Number means there wasn't a gene name associated with the Accession number

Fix: Instead of a number, replace with "NULL".

Dates show up due the excel built in properties (i.e. SEPT2 will become 9/2/2013).

Fix: Change cell type to "Text" on the "Home" tab, then manually type the gene in again.

Cutoff

-Select the expression columns and highlight cells that have values <100,000.

-All values that are highlighted, replace the values with "0"

Normalization

Values were based on their BSA and by how much starting protein.

Clustering

-Go to http://genepattern.broadinstitute.org/gp/pages/index.jsf

-Cluster -> Hierarchical Clustering\

-Used the following settings:

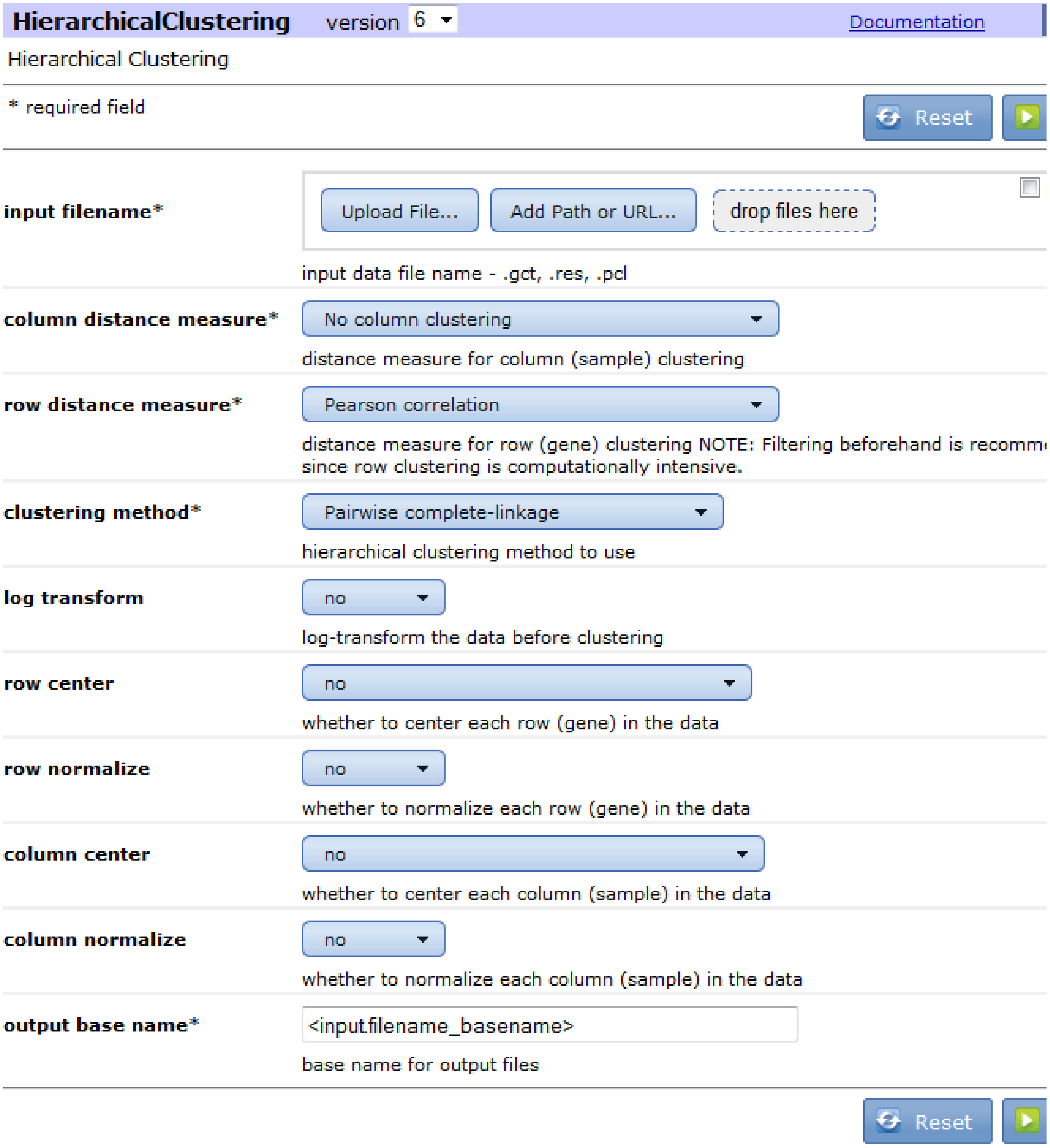

-To set up the file for clustering, use the following image as a guide:

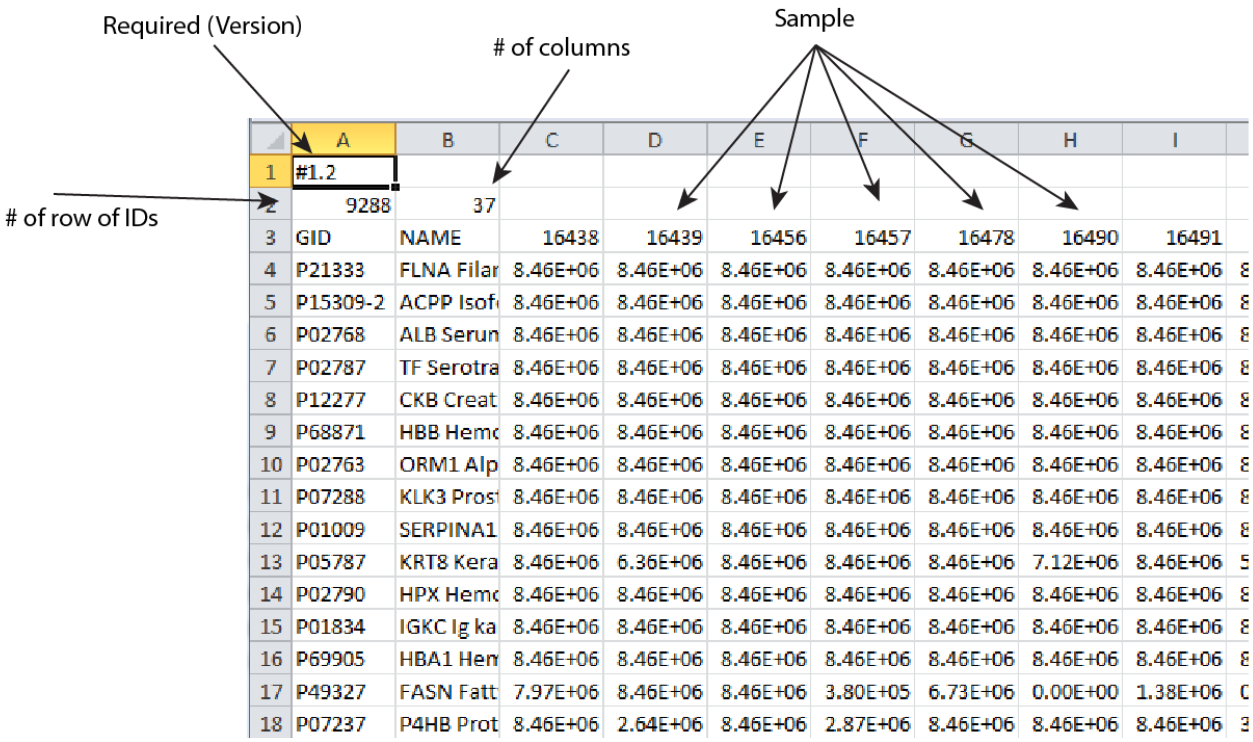

	A	B	C	D	E	F	G	H	I	
1	#1.2									
2	9288	37								
3	GID	NAME	16438	16439	16456	16457	16478	16490	16491	
4	P21333	FLNA Filar	8.46E+06	8.46E+06	8.46E+06	8.46E+06	8.46E+06	8.46E+06	8.46E+06	8
5	P15309-2	ACPP Isof	8.46E+06	8.46E+06	8.46E+06	8.46E+06	8.46E+06	8.46E+06	8.46E+06	8
6	P02768	ALB Serun	8.46E+06	8.46E+06	8.46E+06	8.46E+06	8.46E+06	8.46E+06	8.46E+06	8
7	P02787	TF Serotra	8.46E+06	8.46E+06	8.46E+06	8.46E+06	8.46E+06	8.46E+06	8.46E+06	8
8	P12277	CKB Creat	8.46E+06	8.46E+06	8.46E+06	8.46E+06	8.46E+06	8.46E+06	8.46E+06	8
9	P68871	HBB Hemc	8.46E+06	8.46E+06	8.46E+06	8.46E+06	8.46E+06	8.46E+06	8.46E+06	8
10	P02763	ORM1 Alp	8.46E+06	8.46E+06	8.46E+06	8.46E+06	8.46E+06	8.46E+06	8.46E+06	8
11	P07288	KLK3 Pros	8.46E+06	8.46E+06	8.46E+06	8.46E+06	8.46E+06	8.46E+06	8.46E+06	8
12	P01009	SERPINA1	8.46E+06	8.46E+06	8.46E+06	8.46E+06	8.46E+06	8.46E+06	8.46E+06	8
13	P05787	KRT8 Kera	8.46E+06	6.36E+06	8.46E+06	8.46E+06	8.46E+06	7.12E+06	8.46E+06	5
14	P02790	HPX Hemc	8.46E+06	8.46E+06	8.46E+06	8.46E+06	8.46E+06	8.46E+06	8.46E+06	8
15	P01834	IGKC Ig ka	8.46E+06	8.46E+06	8.46E+06	8.46E+06	8.46E+06	8.46E+06	8.46E+06	8
16	P69905	HBA1 Hen	8.46E+06	8.46E+06	8.46E+06	8.46E+06	8.46E+06	8.46E+06	8.46E+06	8
17	P49327	FASN Fatt	7.97E+06	8.46E+06	8.46E+06	3.80E+05	6.73E+06	0.00E+00	1.38E+06	C
18	P07237	P4HB Prot	8.46E+06	2.64E+06	8.46E+06	2.87E+06	8.46E+06	8.46E+06	8.46E+06	3

-GID contains the accession numbers

-NAME contains the gene name and the description

-To save: File->”Save As” -> File name: “example.gct”(must be exactly like this) -> Save as type: “Text (Tab delimited)” -> “Save”

-Here is an example:

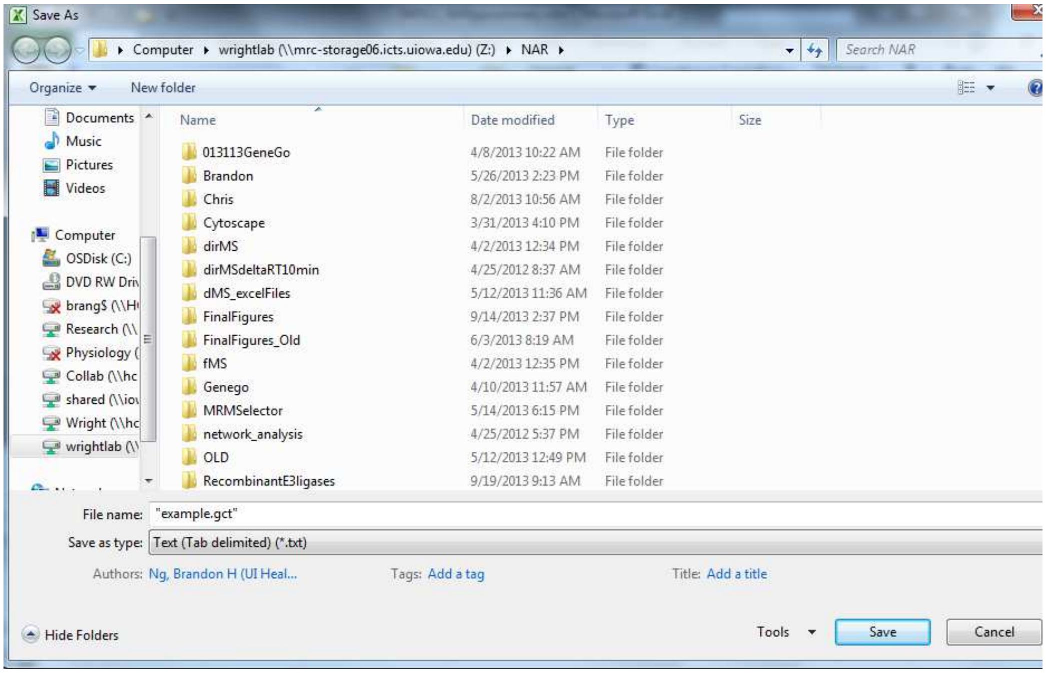

Note: The file name cannot have symbols or uppercased letters

Samples that had the same amount of starting material were placed in the same cluster (i.e. 100, 50, <20)

Supplemental Figure 1A. **Androgen-mediated proliferation of LNCaP prostate-tumor cells is dose-dependent.** Tissue-culture plates (96-well) were seeded with LNCaP cells in androgen-depleted (*AD*) growth medium (phenol-red deficient RPMI1640 containing 10% charcoal-stripped serum) for 24 hrs and challenged with vehicle (EtOH; 0 nM R1881) and different concentrations of synthetic androgen R1881 (*i.e.*, 0.1, 1, or 10 nM). The relative fluorescence units (RFUs) of vehicle- and androgen-treated LNCaP cells were determined at 48 hrs (*i.e.*, day 2), 96 hrs (*i.e.*, day 4), and 144 hrs (*i.e.*, day 6) after the challenge, using *AD* growth medium with the CyQUANT Cell Proliferation Assay Kit. The mean and standard deviation RFU values for each experimental condition (12 wells per condition, *i.e.* 12 biological replicates) and timepoint were plotted for a single independent experiment. The colored lines (*i.e.*, brown, 0 nM; purple, 0.1 nM; green,1.0 nM; red,10.0 nM R1881) connect the mean values for each condition at 48, 96, and 144 hrs.

Supplemental Figure 1B. **Androgen-mediated proliferation of LNCaP prostate-tumor cells is dose-dependent.** Tissue-culture plates (96-well) were seeded with LNCaP cells in androgen-depleted (*AD*) growth medium (phenol-red deficient RPMI1640 containing 10% charcoal-stripped serum) for 24 hrs and challenged with vehicle (EtOH; 0 nM R1881) and different concentrations of synthetic androgen R1881 (*i.e.*, 0.1, 1, or 10 nM). The relative fluorescence units (RFUs) of vehicle- and androgen-treated LNCaP cells were determined at 48 hrs (*i.e.,* day 2), 96 hrs (*i.e.*, day 4), and 144 hrs (*i.e.*, day 6) after the challenge, using *AD* growth medium with the CyQUANT Cell Proliferation Assay Kit. The mean and standard deviation RFU values for each experimental condition (12 wells per condition, *i.e.*, 12 biological replicates) and timepoint were plotted for a single independent experiment. The colored lines (*i.e.*, brown, 0 nM; purple, 0.1 nM; green,1.0 nM; red,10.0 nM R1881) connect the mean values for each condition at 48, 96, and 144 hrs.

Supplemental Figure 2. **Uncropped western blot analyses of proteins extracted from LNCaP cells shown in Figures 1 and 5.** Red boxes denote cropped regions of the scanned western blots using Adobe Photoshop version CS6 software.

Supplemental Figure 3. **Network visualization of enriched pathways in androgen-responsive glycoproteins in LNCaP cells.** Protein interaction networks were built in Cytoscape, with ontology and annotation information downloaded through the Cytoscape interface. Plots for 0 nM, 0.1 nM, 1.0 nM, and 10.0 nM R1881 are shown. Detailed descriptions of the steps used in the processing and filtering of data are provided in the supplemental methods section below.

Supplemental Figure 4. Dose-dependent theoretical clusters. *K*-means clustering of 27 theoretical cluster graphs of androgen-regulated glycoproteins in LNCaP cells.

Supplemental Figure 5. *K* Means Clustering of lectin-enriched proteins in LNCaP prostate tumor cells. The 27 protein clusters generated using the K Means Clustering algorithm. Protein abundance values for 0, 0.1, 1.0, and 10.0 nM R1881 samples were uploaded into RStudio.

Supplemental Figure 6. Theoretical clusters associated with 10 nM R1881 growth-inhibition. Concordant and discordant theoretical cluster graphs associated with 10 nM growth-inhibition.

Supplemental Figure 7. Hematoxylin and eosin visualization of clinical samples. H&E images of normal adjacent tissue (NAT), benign prostate hyperplasia (BPH), localized prostate cancers (CaP), and metastatic prostate cancers (mPCa) as visualized under a light microscope at 10X. Slides contained vendor ID numbers for Proteogenex, University of Iowa Tissue Core, and BioServices. Other

sample identifiers included age of patient, tissue type, tissue weight, Gleason score, PSA level, cancer stage, and percent tumorous tissue, if available.

Supplemental Figure 8. Silver stain analyses of proteins extracted from clinical tissues. For samples R01388233–16969 were loaded in even numbered lanes with each sample loaded with 1 µl, whereas odd numbered lanes were loaded with 2 µl of sample. For all other samples, 1 µl of sample was loaded in lanes 5, 8, and 11, 2 µl of sample was loaded in lanes 6, 9, and 12; and 4 µl of sample was loaded for lanes 7, 10, and 13. LNCaP whole-cell lysate were used as a control and were loaded according to the following amounts: well 1 = 0.5 µg, well 2 = 1 µg, well 3 = 2 µg, and well 4 = 4 µg. The final protein concentration of each sample was estimated by comparing the staining intensity of silver stained gels loaded with control lysates, using the NIH ImageJ software program.

Supplemental Figure 9. Silver stain analysis of lectin-enriched glycoproteins from clinical prostate tissues. Control lysates (LNCaP total protein) were loaded according to the following quantities: well 1 = 0.5 µg, well 2 = 1 µg, well 3 = 2 µg, and well 4 = 4 µg. Odd-numbered sample lanes were loaded with 1 µl whereas even-numbered lanes were loaded with 2 µl. The final protein concentration for each sample was estimated by comparing the staining intensity of silver stained gels loaded with control lysates, using the NIH ImageJ software program.

Supplemental Figure 10. Protein identifications in clinical prostate tissues. Summary of non-redundant protein IDs in NAT, BPH, localized PCa, and metastatic PCa tissue samples as determined by directed MS (dMS) analyses on the Agilent 6520 Accurate-Mass Quadropole Time-of-Flight (Q-TOF) mass spectrometer. The number of shared and unique protein IDs for each tissue sample are shown.

Supplement Figure 11. Androgen-regulated expression of ENTPD5 in LNCaP prostate tumor cells. LNCaP cells grown for 72 hrs in AD growth medium were exposed to vehicle (i.e., EtOH) or the synthetic androgen R1881 at 0.1, 1, and 10 nM for 24 hrs. Total RNA was extracted from vehicle and androgen-treated cells using the RNeasy Midi Kit. First-strand cDNA synthesis was performed with the SuperScript® III First-Strand Synthesis kit, and real-time quantitative PCR was performed with the SYBR Green PCR Master Mix, using qPCR primers directed to GADPH, AR, and ENTPD5. Normalized Ct values in experimentally-treated samples at .1, 1, and 10 nM R1881 were determined based upon GAPDH, KLK3, and ENTPD5 gene expression values in vehicle-treated samples using the Ct method.

Supplemental Figure 12. Characterization of androgen receptor ChIP-Seq peaks at *ENTPD5* in LNCaP cells. (**A**) The ChIP-Seq file (GSM353644_jy10s123.allregions.txt.gz) from Yu *et al*. was uploaded into Integrated Genome Viewer (IGV) and ChIP-seq signals for ENTPD5 (ranging from 0-150 maximum) counts from the ChIP-Seq experiment were visualized. ChIP signals for *ENTPD5* were detected at intron #2 (chr14:74,482,139- 74,482,531) and intron #6 (chr14:74,458,755- 74,459,147). A consensus motif CCASBANNYCCAGCY was detected in introns #2 and #6. The reverse complement motif shows weak conservation to AR motifs M00481: GGWACRNNNTGTNCY and M01201: GGNACRNNRTGTDCT annotated at MotifMap[1,2]. (**B**) The TomTom motif comparison tool was used to compare the consensus motif CCASBANNYCCAGCY to known DNA transcription factor motifs. Three motifs that have significant overlap with the consensus motif CCASBANNYCCAGCY were identified. This included an ELK4 motif (MA0076.2), an ETS1 full motif, and an LBX2_DBD_1 motif.

Supplemental Figure 13. Steps involved in the hierarchical clustering of glycoproteins in LNCaP cells and clinical prostate tissue samples. A detailed description of how the glycoproteomic datasets were uploaded and analyzed with the GenePattern software program hosted at the Broad Institute, Boston MA[3].

SUPPLEMENTAL METHODS SECTION:

***K* means clustering of lectin-enriched glycoproteins in LNCaP cells:** The 27 theoretical protein clusters were obtained by excluding any protein/row that had a 0 value for the measured abundance. All abundance values of the remaining 717 proteins/rows were normalized. The normalization was performed on every protein by dividing each of the 4 abundance values (*i.e.* 0 nM, 0.1 nM, 1.0 nM, and 10 nM R1881) by their total sum. The normalized values were exported from Excel in a tab-delimited text file with five columns and header row: accession, 0 nM, 0.1 nM, 1 nM and 10 nM. The file was loaded into RStudio using the command dataArray <- read.table("ghd-data.txt", row.names=1, header=TRUE, sep="\t") and the resulting variable name "dataArray", as stored input normalized data. The command "row.names=1" told the program that the first column of protein names were row names and the command "header=TRUE" told the program that the file had column headers. The command sep="\t" specified tabs as the delimiter. Once the data were loaded, K means clustering was executed for 27 clusters using the command: dataCluster <- kmeans(dataArray, 27, nstart=20). This stored the clustered results into the variable dataCluster, and the last "nstart=20" specifies trying 20 random starting assignments and choosing the one with the lowest within cluster variation. Clustered data was written out to a results file using the following commands: outputClusters <- dataCluster$cluster write.csv(outputClusters, file="clusters.csv"). The "clusters.csv" file contains two columns; the first is the protein accession number and the second is the cluster value as an integer. The output was imported back into Excel, the appropriate cluster was pasted into the spreadsheet (using the VLOOKUP command), the normalized data were sorted by clusters, and each cluster was plotted separately.

Discovery of DNA motifs in ENTPD5 intron 2 and 6: (Supplemental Figure 10A)-The MEME program was used to identify DNA sequence motifs on ChIP-seq peaks at the *ENTPD5* locus. ChIP-seq signals present in the GEO sample accession: GSM35364 (*i.e.*, GSM353644_ jy10s123.allregions.txt.gz), which contained enriched DNA signals bound by the AR in R1881-treated LNCaP cells, was retrieved from human reference genome (hg19/GRCh37) with Integrated Genomics Viewer (IGV) program. A ChIP signal maximum threshold was selected for 150 counts to highlight ChIP signals in ENTPD5 intron 2, which encompassed 375 base pairs, and ENTPD5 intron 6, which encompassed 125 base pairs, were used for MEME analyses[4]. The Fasta formatted DNA sequences were uploaded into MEME and searched against motifs of Human DNA using the HOCOMOCO Human (v11 CORE) database. The 15-base pair CCASBANNYCCAGCY motif was the longest and most frequently detected (seven sites) consensus motif in introns #2 and #6 of *ENTPD5*.

Tomtom motif comparison of CCASBANNYCCAGCY motif in *ENTPD5*: The 15-bp CCASBANNYCCAGCY motif was searched against motifs of Human DNA using the HOCOMOCO Human (v11 CORE) database using the Tomtom Motif Comparison Tool program[5]. Motifs showing significant overlap with CCASBANNYCCAGCY were detected and displayed in the output (Supplemental Figure 10B).

Protocol for network visualization of lectin-enriched proteome in LNCaP cells: Proteins identified in vehicle- and androgen-treated samples were uploaded into Cytoscape 3.1[6]. Protein interaction networks were built in Cytoscape, with ontology and annotation information downloaded through the Cytoscape interface using the PINA4MS 2.0 plugin[7]. The enriched network output was then annotated and organized within the software platform to visualize the results. The enriched networks were color-coded, with the larger nodes indicating higher statistically significant enrichment value and smaller nodes indicating smaller statistically significant enrichment value. The -log(p-value) statistical significance ranged from 0 to 5.

Protocol for Cytoscape visualization of STRING protein-protein interaction graphs: (Figure 2C)- The list of genes from the 10 nM growth-inhibited clusters (Figure 2B) (Supplemental Excel file 3) were analyzed in the Cytoscape version 3.7.1 software program and subjected to STRING protein query analysis using the Human species database with a confidence score of .4. The output protein-protein interactions were visualized with the perfuse force directed layout and manipulated so that edges could be observed between PPIs. All PPI graphs shown in Figure 2C contained PPI enrichment scores < 0.05, demonstrating that PPI graphs contained more protein interactions between the group of proteins than would be expected from a random set of proteins of similar size drawn from the human genome[6].

Protocol for WebGestalt overrepresentation enrichment analysis (ORA) of 10 nM growth-inhibition clusters: (Figure 2C)- The list of genes from the 10 nM growth-inhibited clusters (Figure 2B) (Supplement Excel X) were uploaded into the WebGestalt software program and analyzed for ORA[8,9]. The settings included Homo sapiens as the organism of interest, geneontology for functional database, and genome encoding-protein as the reference gene list. PPI graphs were constructed in Cytoscape for growth-inhibition clusters with FDR and P values ≤ 0. Only clusters 2, 5, 7, 22, and 26 passed this threshold and PPI graphs displayed in Figure 2C.

References:

1 Xie, X., Rigor, P. & Baldi, P. MotifMap: a human genome-wide map of candidate regulatory motif sites. *Bioinformatics* **25**, 167-174, doi:10.1093/bioinformatics/btn605 (2008).

2 Daily, K., Patel, V. R., Rigor, P., Xie, X. & Baldi, P. MotifMap: integrative genome-wide maps of regulatory motif sites for model species. *BMC Bioinformatics* **12**, 495, doi:10.1186/1471-2105-12-495 (2011).

3 Reich, M. *et al.* GenePattern 2.0. *Nature Genetics* **38**, 500-501, doi:10.1038/ng0506-500 (2006).

4 Bailey, T. L. & Elkan, C. Fitting a mixture model by expectation maximization to discover motifs in biopolymers. Report No. CONF-9408117-; Other: CNN: Grant HG00005; TRN: 96:005197-0004 United States Other: CNN: Grant HG00005; TRN: 96:005197-0004 American Association for Artificial Intelligence, 445 Burgess Drive, Menlo Park, CA 94025 (United States) OSTI English, Medium: X; Size: pp. 28-36 (; Stanford Univ., CA (United States), 1994).

5 Gupta, S., Stamatoyannopoulos, J. A., Bailey, T. L. & Noble, W. S. Quantifying similarity between motifs. *Genome Biology* **8**, R24, doi:10.1186/gb-2007-8-2-r24 (2007).

6 Shannon, P. *et al.* Cytoscape: A Software Environment for Integrated Models of Biomolecular Interaction Networks. *Genome Res.* **13**, 2498-2504 (2003).

7 Cowley, M. J. *et al.* PINA v2.0: mining interactome modules. *Nucleic Acids Research* **40**, D862-D865, doi:10.1093/nar/gkr967 (2011).

8 Zhang, B., Kirov, S. & Snoddy, J. WebGestalt: an integrated system for exploring gene sets in various biological contexts. *Nucleic Acids Res* **33**, W741-W748, doi:10.1093/nar/gki475 (2005).

9 Wang, J., Duncan, D., Shi, Z. & Zhang, B. WEB-based GEne SeT AnaLysis Toolkit (WebGestalt): update 2013. *Nucleic Acids Res* **41**, W77-83, doi:10.1093/nar/gkt439 (2013).

Supplemental Table I: Mass Spectrometry Statistics

R1881 (nM)	0	0.1	1	10
Number of LC-MS/MS runs	9	9	9	9
Total MS/MS spectra	63,728	61,346	63,128	64,277
Number of distinct peptides	5,499	5,393	5,046	5,461
Number of distinct proteins (FDR < 1%, SpectrumMill)	1,540	1,558	1,668	1,586

Supplemental Table II: Clinical Features of Tissue Samples

Summary of Clinical Samples		
**Characteristic**	**# out of 37**	**% total**
NAT/BPH	18	49
Localized T2	13	35
Mets	6	16
**Non-cancerous Samples**		
**Characteristic**	**# out of 18**	**% total**
NAT	8	44
BPH	10	56
**Cancerous Samples**		
**Characteristic**	**# out of 19**	**% total**
Gleason 6	3	16
Gleason 7	11	58
Gleason 9	3	16
Gleason 10	1	5
N/A	1	5
Stage II	8	42
Stage III	8	42

Supplemental Table III: Clinical features of tissue samples

Normal Adjacent Tissue (NAT) and Benign Prostate Hyperplasia (BPH) Samples

No.	Sample ID	Patient Age	PSA	Gleason Score	Cancer Stage	% Tumor	Sample	Vendor
1	16456T1(1)	61	N/A	—	—	—	BPH	ProteoGenex
2	16457T1(2)	67	N/A	—	—	—	BPH	ProteoGenex
3	16478B1(2)	61	N/A	—	—	—	BPH	ProteoGenex
4	16490T1(4)	60	N/A	—	—	—	BPH	ProteoGenex
5	16491T1(6)	61	N/A	—	—	—	BPH	ProteoGenex
6	16494T1(2)	65	N/A	—	—	—	BPH	ProteoGenex
7	16557T1(2)	60	N/A	—	—	—	BPH	ProteoGenex
8	16559T1(2)	66	N/A	—	—	—	BPH	ProteoGenex
9	16438T1(2)	66	N/A	—	—	—	BPH	ProteoGenex
10	16439T1(2)	60	N/A	—	—	—	BPH	ProteoGenex
11	16743T1	62	N/A	—	—	—	NAT	ProteoGenex
12	16769T1	60	N/A	—	—	—	NAT	ProteoGenex
13	16884T1	61	N/A	—	—	—	NAT	ProteoGenex
14	16923T1	65	N/A	—	—	—	NAT	ProteoGenex
15	16929T1	54	N/A	—	—	—	NAT	ProteoGenex

Localized Cancer Samples

No.	Sample ID	Patient Age	PSA	Gleason Score	Cancer Stage	% Tumor	Sample type	Vendor
16	16743T2	62	N/A	3+4=7	STAGE III	60	T2 -	ProteoGenex
17	16769T2	60	N/A	3+3=6	STAGE III	70	T2 -	ProteoGenex
18	16884T2	61	N/A	3+4=7	STAGE II	100	T2 -	ProteoGenex
19	16923T2	65	N/A	4=3=7	STAGE III	100	T2 -	ProteoGenex
20	16929T2	54	N/A	3=4=7	STAGE III	75	T2 -	ProteoGenex
21	R90197381	61	7.77	5+5=10	N/A	N/A	T2 -	UI Tissue Core
22	6TQGJ	71	N/A	4+3=7	STAGE II	80	T2 -	BioServe
23	GT55Q	66	N/A	3+3=6	STAGE II	80	T2 -	BioServe
24	IPUUV	63	N/A	3+4=7	STAGE II	55	T2 -	BioServe
25	Q7ZK7	69	N/A	3+4=7	STAGE II	80	T2 -	BioServe
26	R03188233	N/A	5.9	3+4=7	N/A	N/A	T2 -	UI Tissue Core
27	R42521246	70	37.43	4+5=9	N/A	15	T2 -	UI Tissue Core
28	R67820304	51	7.18	4+3=7	N/A	N/A	T2 -	UI Tissue Core
29	R93776568	N/A	4.08	3+4=7	N/A	N/A	T2 - Localized	UI Tissue Core

Metastatic Cancer Samples

No.	Sample ID	Patient Age	PSA	Gleason Score	Cancer Stage	% Tumor	Sample	Vendor
30	6FOOW	61	N/A	4+3=7	STAGE III	55	Met (Colon)	BioServe
31	IVC2L	72	N/A	3+3=6	STAGE III	65	Local Met	BioServe
32	V6KBD	71	N/A	5+4=9	STAGE III	90	Local Met	BioServe
33	OUULN	60	N/A	3+4=7	STAGE IV	50	Met (Bladder, Neck)	BioServe
34	A7KW3	82	N/A	N/A	NA	95	Met (Brain)	BioServe
35	D1VAB	62	N/A	4+5=9	STAGE IV	80	Met (Lymph Node)	BioServe

The yellow highlighted tissue samples 16769T1 and R67820304 were lost during sample processing and excluded from the glycoproteomic analyses.

Supplemental Table IV: Protein quantification of Normal Adjacent Tissue & Benign Prostate Hyperplasia

Sample ID	Sample Weight	Weight Used	Extract Concent.	Buffer Used	Total Protein	Glyco-enrichment	Post-dialysis	Total Protein	Protein Retention	Amount used for proteomics
16743T1c2	0.83 g	0.83 g	3 ug/ul	10 ml	30 mg	11.5 mg	.59 ug/ul	1416 ug	12.30%	50 ug
16769T1c1	0.68 g	0.68 g	1.5 ug/ul	8 ml	12 mg	11.5 mg	.89 ug/ul	1958 ug	17%	50 ug
16884T1c1	0.44 g	0.44 g	3 ug/ul	5 ml	15 mg	11.5 mg	.79 ug/ul	1817 ug	15.80%	50 ug
16923T1c1	0.44 g	0.44 g	3 ug/ul	5 ml	15 mg	11.5 mg	.84 ug/ul	1848 ug	16.10%	50 ug
16929T1c1	0.79 g	0.79 g	3 ug/ul	10 ml	30 mg	11.5 mg	.33 ug/ul	726 ug	6.30%	50 ug
16438T1c2	0.56 g	0.56 g	1.5 ug/ul	8 ml	12 mg	10 mg	0.5 ug/ul	1000 ug	10%	100 ug
16439T1c2	0.511 g	0.511 g	2 ug/ul	8 ml	16 mg	10 mg	0.6 ug/ul	1200 ug	12%	100 ug
16456T1c1	0.809 g	0.809 g	3 ug/ul	8 ml	24 mg	10 mg	0.7 ug/ul	1400 ug	14%	100 ug
16457T1c2	0.819 g	0.819 g	2.5 ug/ul	8 ml	20 mg	10 mg	0.7 ug/ul	1400 ug	14%	100 ug
16478B1c2	0.723 g	0.723 g	1.5 ug/ul	8 ml	12 mg	10 mg	0.5 ug/ul	1000 ug	10%	100 ug
16490T1c4	0.793 g	0.793 g	4 ug/ul	8 ml	32 mg	10 mg	0.5 ug/ul	1000 ug	10%	100 ug
16791T1c6	0.606 g	0.606 g	3.5 ug/ul	8 ml	28 mg	10 mg	0.6 ug/ul	1200 ug	12%	100 ug
16494T1(2)c	0.602 g	0.602 g	2.5 ug/ul	8 ml	20 mg	10 mg	0.55 ug/ul	1100 ug	11%	100 ug
16557T1c2	0.0851 g	0.0851 g	4 ug/ul	8 ml	32 mg	10 mg	0.75 ug/ul	1500 ug	15%	100 ug
16559T1c(2)	0.656 g	0.656 g	3.5 ug/ul	8 ml	28 mg	10 mg	0.85 ug/ul	1700 ug	17%	100 ug
R03188233N	NK	2.92 g	0.375 ug/ul	4 ml	1500 ug	800 ug	0.375 ug/ul	70 ug	8.75%	20 ug
R42521246N	59 g	1.59 g	0.25 ug/ul	2 ml	500 ug	400 ug	0.5 ug/ul	25 ug	6.25%	10 ug
R67820304N	44.65 g	1.55 g	3 ug/ul	2 ml	6000 ug	400 ug	0.0125 ug/ul	10 ug	2.50%	8 ug
R90197381N	45.6 g	1.53 g	0.5 ug/ul	1 ml	500 ug	400 ug	0.25 ug/ul	30 ug	7.50%	20 ug
R93776568N	39.55 g	2.28 g	0.375 ug/ul	3 ml	1125 ug	400 ug	0.75 ug/ul	60 ug	15%	20 ug

Localized

Sample ID	Sample Weight	Weight Used	Extract Concent.	Buffer Used	Total Protein	Glyco-enrichment	Post-dialysis	Total Protein	Protein Retention	Amount used for proteomics
16884T2c2	0.40 g	0.40 g	3 ug/ul	5 ml	15 mg	11.5 mg	.68 ug/ul	1632 ug	14.20%	50 ug
GT55Q	0.075g	0.075 g	1 ug/ul	5 ml	5 mg	2 mg	0.15 ug/ul	450 ug	22.50%	100 ug
IPUUV	0.468g	0.468 g	2 ug/ul	8 ml	16 mg	8 mg	0.5 ug/ul	1000 ug	12.50%	100 ug
6TQGJ	0.116 g	0.116 g	1 ug/ul	5 ml	5 mg	5 mg	0.2 ug/ul	500 ug	10%	100 ug
Q7ZK7	0.076 g	0.076 g	0.6 ug/ul	5 ml	3 mg	2 mg	0.15 ug/ul	375 ug	18.80%	100 ug
R03188233T	NK	2.91 g	0.375 ug/ul	4 ml	1500 ug	800 ug	0.5 ug/ul	90 ug	11.25%	20 ug
R42521246T	59 g	3.08 g	0.5 ug/ul	4 ml	2000 ug	400 ug	0.5 ug/ul	35 ug	8.75%	10 ug
R67820304T	44.65 g	1.57 g	1.5 ug/ul	2 ml	3000 ug	400 ug	0.025 ug/ul	23.5 ug	5.90%	8 ug
16743T2c2	0.82 g	0.82 g	3 ug/ul	10 ml	30 mg	11.5 mg	.67 ug/ul	1407 ug	12.20%	50 ug
16769T2c1	0.87 g	0.87 g	1.5 ug/ul	10 ml	15 mg	11.5 mg	.92 ug/ul	2024 ug	17.60%	50 ug
16923T2c1	0.43 g	0.43 g	3 ug/ul	5 ml	15 mg	11.5 mg	1.11 ug/ul	2442 ug	21.20%	50 ug
16929T2c1	0.52g	0.52 g	3 ug/ul	5 ml	15 mg	11.5 mg	1.06 ug/ul	2332 ug	20.30%	50 ug
R90197381T	45.6 g	1.53 g	0.225 ug/ul	1 ml	225 ug	400 ug	0.5 ug/ul	40 ug	10%	20 ug
R93776568T	39.55 g	2.29 g	0.375ug/ul	3 ml	1125 ug	400 ug	0.5 ug/ul	60 ug	15%	20 ug

Metastatic

Sample ID	Sample Weight	Weight Used	Extract Concent.	Buffer Used	Total Protein	Glyco-enrichment	Post-dialysis	Total Protein	Protein Retention	Amount used for proteomics
6FOOW	0.308 g	0.308 g	3 ug/ul	5 ml	15 mg	10 mg	0.3 ug/ul	600 ug	6%	100 ug
A7KW3	0.804 g	0.804 g	4.5 ug/ul	8 ml	36 mg	10 mg	0.3 ug/ul	600 ug	6%	100 ug
D1VAB	0.359 g	0.359 g	2.5 ug/ul	5 ml	12.5 mg	10 mg	0.4 ug/ul	800 ug	8%	100 ug

IVC2L	0.129 g	0.129 g	2 ug/ul	5 ml	10 mg	8 mg	0.2 ug/ul	500 ug	6.25%	100 ug
OUULN	0.25 g	0.25 g	2 ug/ul	5 ml	10 mg	8 mg	0.35 ug/ul	875 ug	10.90%	100 ug
V6KBD	0.198 g	0.198 g	3 ug/ul	5 ml	15 mg	10 mg	0.25 ug/ul	625 ug	6.25%	100 ug

Supplemental Table Legend: Proteins used and retained during processing. Column 1 details the total amount of protein extracted from the tissue samples. Column 2 explains the amount of protein used for glycoprotein enrichment. Columns 3 and 4 list concentrations after dialysis and the approximate total amount of protein recovered, respectively. Retained protein (target glycoproteins of interest) is shown in the second to last column. The final column details the amount of protein digested and further process for mass spec analysis.
